# Cost-effectiveness of bariatric surgery and non-surgical weight management programmes for adults with severe obesity: a decision analysis model

**DOI:** 10.1038/s41366-021-00849-8

**Published:** 2021-06-04

**Authors:** D. Boyers, L. Retat, E. Jacobsen, A. Avenell, P. Aveyard, E. Corbould, A. Jaccard, D. Cooper, C. Robertson, M. Aceves-Martins, B. Xu, Z. Skea, M. de Bruin, and the REBALANCE team, E. Jacobsen, D. Boyers, D. Cooper, L. Retat, P. Aveyard, Fiona Stewart, Graeme MacLennan, Laura Webber, E. Corbould, B. Xu, A. Jaccard, Bonnie Boyle, Eilidh Duncan, Michal Shimonovich, Cynthia Fraser, Lara Kemp

**Affiliations:** 1 Health Economics Research Unit, University of Aberdeen, Aberdeen, UK; 2 UK Health Forum, London, UK; 3 Health Services Research Unit, University of Aberdeen, Aberdeen, UK; 4 Nuffield Department of Primary Care Health Sciences, Oxford University, Oxford, UK; 5 NIHR Oxford Biomedical Research Centre (BRC) Obesity, Diet and Lifestyle Theme, Oxford, UK; 6 NIHR Applied Research Collaboration (ARC) Oxford and Thames Valley, Oxford, UK; 7 Health Psychology, University of Aberdeen, Aberdeen, UK; 8 Radboud University Medical Center, Radboud Institute for Health Sciences, IQ Healthcare, Nijmegen, The Netherlands

**Keywords:** Weight management, Lifestyle modification, Health policy

## Abstract

**Objectives:**

To determine the most cost-effective weight management programmes (WMPs) for adults, in England with severe obesity (BMI ≥ 35 kg/m^2^), who are more at risk of obesity related diseases.

**Methods:**

An economic evaluation of five different WMPs: 1) low intensity (WMP1); 2) very low calorie diets (VLCD) added to WMP1; 3) moderate intensity (WMP2); 4) high intensity (Look AHEAD); and 5) Roux-en-Y gastric bypass (RYGB) surgery, all compared to a baseline scenario representing no WMP. We also compare a VLCD added to WMP1 vs. WMP1 alone. A microsimulation decision analysis model was used to extrapolate the impact of changes in BMI, obtained from a systematic review and meta-analysis of randomised controlled trials (RCTs) of WMPs and bariatric surgery, on long-term risks of obesity related disease, costs, quality adjusted life years (QALYs) and incremental cost-effectiveness ratios (ICERs) measured as incremental cost per QALY gained over a 30-year time horizon from a UK National Health Service (NHS) perspective. Sensitivity analyses explored the impact of long-term weight regain assumptions on results.

**Results:**

RYGB was the most costly intervention but also generated the lowest incidence of obesity related disease and hence the highest QALY gains. Base case ICERs for WMP1, a VLCD added to WMP1, WMP2, Look AHEAD, and RYGB compared to no WMP were £557, £6628, £1540, £23,725 and £10,126 per QALY gained respectively. Adding a VLCD to WMP1 generated an ICER of over £121,000 per QALY compared to WMP1 alone. Sensitivity analysis found that all ICERs were sensitive to the modelled base case, five year post intervention cessation, weight regain assumption.

**Conclusions:**

RYGB surgery was the most effective and cost-effective use of scarce NHS funding resources. However, where fixed healthcare budgets or patient preferences exclude surgery as an option, a standard 12 week behavioural WMP (WMP1) was the next most cost-effective intervention.

## Introduction

In England, 26% of adult men and 29% of adult women are obese (BMI ≥ 30 kg/m^2^) [1]. Adults with severe obesity, defined here as having a Body Mass Index (BMI) ≥ 35 kg/m^2^, have substantially increased incidence of cardiovascular disease, stroke, respiratory disease, and cancer, which severely limit quality and length of life [2, 3]. Treatment of obesity related disease leads to substantial cost burden on healthcare payers due to increased risk of hospital admission and increased average length of stay [4]. In 2017, the total economic burden of overweight and obesity in England was ~£16 billion [5].

The UK, like many countries, offers a range of treatment options from cheap short duration, low-intensity weight management programmes costing ~£50/treatment to costly treatments, such as bariatric surgery. The most recent National Institute for Health and Care Excellence (NICE) obesity clinical guidance (CG189), published in 2014, recommends multicomponent weight management programmes (WMPs) [6]. These should include behaviour change strategies to help increase physical activity (30 min of moderate or greater intensity physical activity on five or more days a week) and improve dietary behaviour (suggesting calorie deficits of 600 kcal/day for sustainable weight loss). Very low calorie diets (VLCDs, ≤800 kcal/day) were only recommended for people with a clinically assessed need to lose weight rapidly, for example those scheduled for joint replacement surgery or fertility treatment. VLCDs are undergoing testing for people with recent onset type 2 diabetes mellitus (T2DM) [7]. NICE guidance recommends pharmacological therapies if lifestyle interventions have failed, or weight loss has plateaued [6]. Surgery is only available in the general population for people with a BMI ≥ 40 kg/m^2^, or a BMI ≥ 35 kg/m^2^ for patients who have other significant comorbidities such as (T2DM) and who have previously tried and failed to achieve or maintain adequate weight loss [6].

In the absence of effective obesity prevention strategies, WMPs may help reduce the substantial health, social, societal and economic burden of obesity related disease. We conducted a systematic review of economic evaluations on interventions for severe obesity as part of our National Institute for Health Research (NIHR) funded Review of Behaviour And Lifestyle interventions for severe obesity: AN evidence synthesis (REBALANCE) project [8]. However, we found that no studies comprehensively assessed the relative cost-effectiveness of relevant interventions (WMPs, drug therapies and bariatric surgery) within the same analysis. We then undertook this economic evaluation, as part of that project, to provide evidence to inform the most efficient allocation of scarce UK NHS resources for the management of adults with severe obesity [8].

## Methods

### Interventions and comparators

Five different interventions that generated the best long-term effectiveness evidence (greatest reduction in baseline BMI) from our systematic review of randomised controlled trials (RCTs), and in consultation with clinical experts and patient advisors were included in the model [8]. Full details of the intervention content for each contributing study are provided in our full report and appendices and described briefly below [8].

#### Weight management programme (WMP1)

WMP1 represents short duration low intensity behavioural programmes, typically delivered in one-hour long group sessions over ~12 weeks, offering advice on diet and physical activity. WMP1 is similar in content to NICE based recommendations for initial weight management as part of Tier 2 services, with follow-up [6].

#### VLCDs added to WMP1

VLCDs were defined as meal replacement products that replace meals and provide up to 800 kcal/day (±10%). VLCDs were provided in addition to low-intensity WMPs as defined under WMP1.

#### WMP2

WMP2 was a medium intensity lifestyle intervention, similar in content to a shortened Look AHEAD study (see below) and the Diabetes Prevention Programme (DPP) from which Look AHEAD was developed, but with shorter duration of intervention delivery, generally over one year [9]. The intervention is typically used as the comparator in RCTs of bariatric surgery.

#### Look AHEAD (Action for HEAlth in Diabetes)

Look AHEAD was a long-term, high intensity intervention delivered in a large US based RCT (5145 participants) that ran from 2001 to 2012, with a median follow-up of 9.6 years. The study was unique in that long-term support to maintain weight loss was provided for the duration of follow-up. The study aimed to determine whether intentional weight loss reduced cardiovascular morbidity and mortality in individuals with T2DM [10, 11]. The Look AHEAD lifestyle intervention provided behavioural support, dietary interventions, and physical activity programmes. The Look AHEAD study provides the most reliable, long term weight loss data for any non-surgical intervention. The study has demonstrated a continued weight loss over the entire trial time follow up period, reduced incidence of obesity related diseases, and reduced hospital days by 15% compared with the control group [11, 12]. There are several publications regarding resource use and cost [12], quality of life [13] and effectiveness in terms of weight loss [14], as well as a recently published within-trial cost-effectiveness analysis of the original Look AHEAD intervention [15].

#### RYGB

RYGB was chosen as the type of bariatric surgery for inclusion in the model because it appears to be the most effective and common type of bariatric surgery undertaken in the NHS [8].

The economic evaluation aimed to address the cost-effectiveness of:

1. Each of the five interventions compared to a baseline scenario (i.e. no intervention), generating five separate pairwise comparisons of cost-effectiveness.
2. All interventions compared against each other in a fully incremental analysis to identify the most cost-effective intervention overall.
3. VLCDs added to WMP1 compared to WMP1 alone, in order to assess the value of adding VLCDs to existing weight management programmes.

#### Baseline scenario

The baseline scenario reflected BMI trends over time in England for the subgroup of the general adult population with BMI ≥ 35 kg/m^2^, with no additional intervention costs. The baseline scenario can be considered similar to the current status quo in the UK, where in general, delivery of weight loss interventions does not occur at scale. The baseline scenario is intended to closely represent a real-world scenario where there are no widely provided weight loss interventions.

### Model description

We used the UK Health Forum’s (UKHF) semi-Markovian microsimulation model to assess the long term health benefits measured in quality adjusted life years (QALYs), NHS perspective costs, and cost-effectiveness (incremental cost per QALY gained) of the different weight management interventions compared to a baseline scenario that represents current standard practice in England [16]. Given the scarcity of delivery of NHS weight loss services at scale for adults with severe obesity, we have considered the baseline, standard care scenario to reflect no routinely delivered weight loss intervention. Full technical details regarding the model specification, structure and parameterisation, including incidence, prevalence and mortality data can be found in Supplementary Material 1.

Briefly, the model simulated a virtual closed cohort (only losing members through death) of 50 million adults with severe obesity (BMI ≥ 35 kg/m^2^) sampled according to age and gender characteristics from the English adult population. The population aged each year, with BMI following national age-sex-BMI specific trends using serial cross-sectional data from the Health Survey for England (HSE) between 2003 and 2014 to create longitudinal projections of the future proportion of the population in different BMI categories, defined by 5-year age groups and sex [17]. Annual mortality rates were obtained from ONS population statistics [18].

When the cohort entered the model, a Monte-Carlo process was used to stochastically apply incident obesity related disease, dependent on age, sex and BMI, using data derived from systematic searches of the literature. The model included the disease states: coronary heart disease (CHD), stroke, hypertension, T2DM, knee osteoarthritis and BMI-related cancers (breast, colorectal, endometrial, oesophageal, pancreatic and renal). In each subsequent annual cycle of the model, each disease could occur, remain, or cause death following a semi-Markov method. The relative risk (RR) of developing each obesity related disease in any given model cycle increased by increasing BMI category and by age. For CHD, stroke, hypertension and knee osteoarthritis, the RRs to populate the model were sourced from the Dynamic Model for Health Impact Assessment (DYNAMO-HIA) World Obesity Federation repository [19]. RRs for the remaining obesity related diseases were obtained from a review of the literature. The RR of developing pancreatic cancer was not available from the literature and was assumed to be equal to the RR of dying from it, based on the low survival rate from pancreatic cancer. Further details of all risk equations used in the model are provided in Supplementary Material 1.

The model predicted the incidence and mortality associated with a range of obesity related diseases according to current and projected future BMI. Assuming a binomial distribution for the incidence, the model computed the mean estimate and variance of disease incidence from the 50 million Monte Carlo trials. Each obesity related disease was associated with direct healthcare cost and utility implications accumulated in annual model cycles over a 30-year time horizon from 2016 to 2046. Interventions were delivered once only, to each eligible member of the population starting at the beginning of 2016. A UK NHS perspective was adopted for the analysis. Costs and benefits occurring beyond the first year were discounted at a rate of 1.5% per annum, in line with NICE’s approach to economic evaluation of public health interventions [20].

### Costs

Costs were included in the model using 2016 unit costs, or inflating older costs to 2016 values where necessary using an online cost converter tool [21]. Costs reflect the resources required to deliver the respective interventions as well the resources required to treat downstream obesity related disease.

### Intervention cost

A component costing approach was used to derive the delivery costs for each intervention. Detailed resource use data were available from the published literature for the Look AHEAD study [12]. Where possible, resources required to deliver the WMP and VLCD interventions were obtained from studies used to generate effectiveness (i.e. BMI change) data [8]. This ensured that the cost and benefit parameters used in the model were obtained from consistent sources. Resource use included staff time, meal replacements, rental of venues (costed at a rate of £50 per hour), provision of materials, and the provision of any supplementary vitamins or minerals. All resource use was costed according to UK national average unit costs. A breakdown of costs for each intervention by year and component of cost is provided in Supplementary Material 2.

For RYGB, costs included pre-operative resource use (time to prepare a patient for surgery), operative costs for RYGB (NHS reference costs for 2015-16, healthcare resource group (HRG) code FZ84Z) and post-discharge resource use informed by previous economic evaluations and intervention descriptions in RCTs [8, 22]. Resource use in the first five years following surgery included outpatient visits, appointments with dietitians and psychologists. From year six onwards, for the remaining duration of the model time horizon, we assumed an annual outpatient visit including consultations with a dietitian, blood test, and vitamin / nutrient supplementation in line with British Obesity and Metabolic Surgery Society (BOMSS) guidelines [23]. Published surgery complication rates were costed as the weighted average of day case and inpatient admissions across the different complication and co-morbidity HRGs for bariatric surgery [24]. The 10-year surgery revision rate was obtained from the SOS (Swedish Obese Subjects) study and costs were assumed equal to the original intervention delivery [25]. Table 1 illustrates the delivery costs for each intervention over time. Full details on the approach used to generate cost and utility implications of surgery complications are provided in Supplementary Material 2.

**Table 1:** Intervention delivery costs by year and scenario (£).

Year	WMP1	VLCD added to WMP1	WMP2	Look AHEAD	RYGB
Year 1	£619	£1893	£754	£2189	£8253
Year 2	£268	£268	£152	£1452	£1559
Year 3	£60	£60	£186	£1270	£921
Year 4	£9	£9	£204	£1092	£659
Year 5	N/A	N/A	£111	£760	£663
Year 6	N/A	N/A	N/A	£760	£619
Year 7	N/A	N/A	N/A	£760	£619
Year 8	N/A	N/A	N/A	£760	£619
Year 9	N/A	N/A	N/A	£760	£619
Year 10	N/A	N/A	N/A	N/A	£619
Annual costs from year 11 to 30	N/A	N/A	N/A	N/A	£536
**Total costs (undiscounted)**	**£956**	**£2230**	**£1407**	**£9804**	**£25,862**

Intervention delivery costs by year and scenario.

*N/A* Not applicable, *RYGB* Roux-en-Y gastric bypass surgery, *WMP1* Weight Management Programme 1, *WMP2* Weight Management Programme 2, *VLCD* Very Low Calorie Diet.

The values in bold indicate the final total intervention costs summed across the full time horizon (i.e. all years together).

### Costs of obesity related disease

The direct NHS perspective healthcare costs per case/year of each obesity related disease were based on a review of the literature. Costs for each obesity related disease comprised hospital inpatient, outpatient, primary care and prescription medications. The use of programme budgeting costs, reflecting annual NHS England expenditure across different disease programmes, was considered but deemed to underestimate the true costs of disease in some settings, and was only considered when no other suitable data existed [26]. Intervention delivery costs were added to the costs of treating obesity related disease for each intervention to obtain total costs.

### Quality of life (Utilities)

In the model, people without any obesity related disease were assigned a utility value of 1. Those who died were assigned a utility value of 0. For each obesity related disease, UK specific EQ-5D utilities were sourced from a review of the literature. When more than one candidate utility source was identified, the most recent / representative and largest sample applicable to the UK population was chosen. For simulations where a patient had multiple diseases, these were assumed independent of one another, and hence a multiplicative utility was applied. Disease specific costs and utilities are reported in Table 2. Further detailed information on cost and utility parameters, including their derivation are provided in Supplementary Material 1.

**Table 2:** Disease specific costs and utilities used in the economic model.

	Costs		Utilities		
Disease	Both sexes	Source	Male	Female	Source
CHD	£2838.70^a^	NHS 2015 – Proposed National Standards and Liu et al. (2002) [45, 46]	0.76	0.76	Laires et al. (2015) [47]
Stroke	£1627.26^b^	Saka et al. (2009) [48]	0.713	0.713	Rivero-Arias et al. (2010) [49]
Hypertension	£493.15^b^	Brilleman et al. (2013) [50]	0.721	0.721	Sullivan et al. (2011) [51]
T2DM	£672.28^a^	Minassian et al. (2012) and Kanavos et al. (2012) [52, 53]	0.661	0.661	Sullivan et al. (2011) [51]
Knee Osteoarthritis	£223.97^b^	Chen et al. (2012) [54]	0.49	0.46	Conner-Spady et al. (2015) [55]
Breast Cancer	£13,295.53^b^	Hall et al. (2015) [56]	N/A	0.749	Sullivan et al. (2011) [51]
Colorectal Cancer	£13,563.22^b^	Hall et al. (2015) [56]	0.676	0.676	Sullivan et al. (2011) [51]
Endometrial Cancer	£2471.21^b^	Pennington et al. (2016) [57]	N/A	0.598	Sullivan et al. (2011) [51]
Oesophageal Cancer	£9568.28^b^	Agus et al. (2013) [58]	0.904	0.904	Sullivan et al. (2011) [51]
Ovarian Cancer	£1408.94^c^	NHS programme budget [26]	N/A	0.848	Sullivan et al. (2011) [51]
Pancreatic Cancer	£5735.93^b^	Laudicello (2011) [59]	0.79	0.79	Romanus et al. (2012) [60]
Renal Cancer	£414.81^c^	NHS programme budget [26]	0.661	0.661	Sullivan et al. (2011) [51]

*CHD* Coronary Heart Disease, *N/A* Not Applicable, *NHS* National Health Service, *T2DM* Type 2 Diabetes Mellitus.

^a^Costs estimated from study using top-down approach.

^b^Costs estimated from study using bottom-up approach.

^c^Costs estimated from programme budgets from 2012 inflated to 2016 values.

### Clinical effectiveness (BMI change data used to populate the economic model)

In this model, estimates of cost-effectiveness for all interventions were affected by the BMI trajectory relative to the general population in England who had a BMI ≥ 35 kg/m^2^ (standard care). Two components affected the long-term BMI trajectory.

The first is the effectiveness of the intervention in terms of BMI change. BMI change data were obtained directly from the Look AHEAD study [11]. BMI change for the remaining interventions was calculated based on our systematic review and meta-analyses of RCTs with duration of follow-up ≥1 y. [8] BMI data were adjusted to account for study drop-outs from the RCTs, with data imputed using the baseline observation carried forward for drop-outs [27]. Where data were not reported at annual intervals, a linear interpolation between time points was assumed. Where weight loss data were reported, but not BMI, we calculated change in BMI assuming the average sex-adjusted height of the general population.

**Fig. 1:**
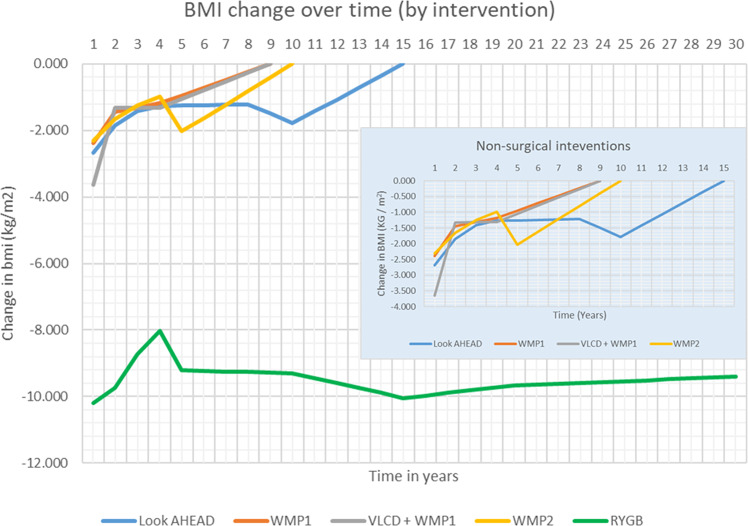
BMI change over time (base-case analysis). details the modelled BMI change over time. The main figure panel includes all modelled interventions, including surgery. The embedded panel details the BMI change modelled for non-surgical WMPs only. Abbreviations: BMI = Body Mass Index; WMP = Weight Management Programme; VLCD = Very low calorie diet; RYGB = Roux-en-Y gastric bypass surgery.

The second is the rate of weight regain to baseline beyond cessation of the intervention. For non-surgical interventions, the base case model assumes a linear return of BMI to baseline over 5 years from the final point that data were reported in the trials, informed by a published meta-analysis of 46 trials [28]. For surgery, 20 year follow up data for gastric bypass patients from the SOS study were used in the model, with an assumption of linear extrapolation out to year 30 [25]. Figure 1 details the changes in BMI over time used to populate the model for each intervention.

### Analysis

Results were reported as incremental cost-effectiveness ratios (ICER). ICERs represent the difference in costs (costs of intervention delivery and obesity related disease) divided by the difference in health outcomes (quality adjusted life years). ICERs were presented for a) pairwise comparisons of each intervention against the population trend and b) incrementally in a multiple-treatment comparison for all candidate interventions, with interventions ranked in ascending order of costs and ICERs reported against the next most effective (in terms of QALY gains), non-dominated ranked intervention.

### Sensitivity analyses

A sensitivity analysis was conducted to reflect the uncertainty surrounding the weight regain assumption applied in the model due to the lack of long-term evidence on this model parameter. Most of the interventions, apart from Look AHEAD and RYGB, had a short trial time horizon, and therefore, exploring the uncertainty regarding what happens after the trial ends in terms of weight change, was most important. The BMI regain was estimated using a linear trend fitted to the available data from the respective studies. Due to the availability of long-term weight regain data, sensitivity analysis did not vary the regain over time for bariatric surgery. Further sensitivity analyses explored the impact on results of varying the discount rate and the model time horizon [29].

## Results

**Fig. 2:**
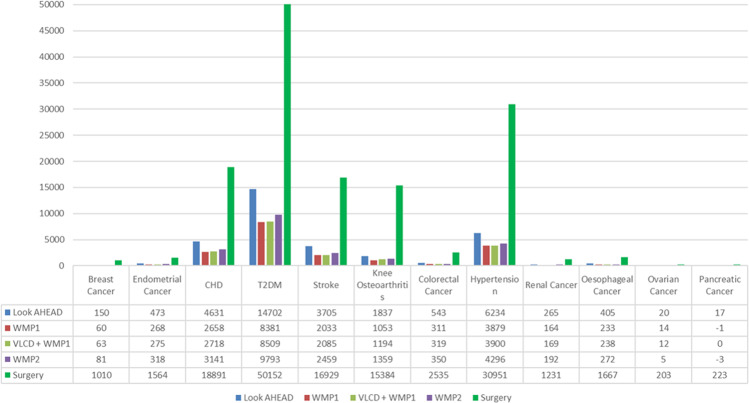
Cumulative incidence cases of obesity related disease avoided per 100,000 population with BMI ≥ 35 KG/M2 compared to population trends. details the cumulative incidence of 12 obesity related disease avoided per 100,000 population with a BMI of 35 and above for each modelled intervention compared to standard care. Abbreviations: BMI = Body Mass Index; WMP = Weight Management Programme; VLCD = Very low calorie diet; RYGB = Roux-en-Y gastric bypass surgery.

The microsimulation model predicted an additional 287,791 [±147] new cumulative instances of obesity related disease, per 100,000 adults with severe obesity by 2046 without population intervention, and many people will develop multiple obesity related diseases. Figure 2 shows that BMI reductions had the greatest impact on future incidence of T2DM, hypertension, stroke and CHD. Due to the superior, sustainable weight loss, RYGB reduces the incidence of obesity related diseases the most, followed by the Look AHEAD intervention. In total, Look AHEAD, WMP1, VLCD added to WMP1, WMP2 and RYGB reduce 32,981; 19,053; 19,482; 22,263 and 140,740 obesity related diseases per 100,000 individuals with severe obesity over a 30-year time horizon.

Table 3 reports the total intervention costs, obesity related disease costs, net impact on total NHS costs, and total QALYs for each modelled intervention. Results are reported for two scenarios: first, assuming a linear 5 year weight regain to baseline for non-surgical WMPs following the end of intervention delivery and secondly, assuming that the rate of weight regain follows the trend of regain observed in the contributing trials. ICERs are reported for each intervention compared to baseline and incrementally against the next most effective (in terms of QALYs) alternative.

**Table 3:** Cost-effectiveness results.

	Intervention cost (£m/100k population)	Obesity disease cost (£m/100k population)	Total cost (£m/100k population)	Total QALY per 100k population	Incremental cost (£m/100K population)	Incremental QALY (per 100K population)	ICER (vs. next best alternative)	ICER (vs. baseline)
Base case analysis								
Baseline	£0	£2898	£2898	1,135,676	–	–	–	–
WMP1	£94	£2814	£2909	1,154,944	£11	19,269	£557	£557
VLCD added to WMP1	£220	£2812	£3032	1,155,963	Dominated	Dominated	Dominated	£6628
WMP2	£135	£2798	£2933	1,158,386	Ext Dom.	Ext Dom.	Ext Dom.	£1540
Look AHEAD	£889	£2754	£3643	1,167,101	Ext Dom.	Ext Dom.	Ext Dom.	£23,725
RYGB Surgery	£2024	£2295	£4319	1,276,038	£1411	121,094	£11,648	£10,126
BMI regain sensitivity analysis								
Baseline	£0	£2898	£2898	1,135,676	Dominated	Dominated	Dominated	–
VLCD added to WMP1	£220	£2840	£3060	1,150,251	Dominated	Dominated	Dominated	£11,152
WMP1	£94	£2834	£2928	1,151,112	Dominated	Dominated	Dominated	£1965
WMP2	£135	£2740	£2875	1,168,178	–	–	–	Dominant
Look AHEAD	£889	£2666	£3555	1,179,771	Ext Dom.	Ext Dom.	Ext Dom.	£14,906
RYGB Surgery	£2024	£2295	£4319	1,276,038	£1444	107,860	£13,392	£10,126

Dominated: An intervention that is both more costly and less effective than a comparator. A dominated intervention doess not offer good value for money and is therefore excluded from the calculation of ICERs.

Ext Dom: Extendedly dominated: An intervention that is excluded because an alternative intervention can deliver greater QALY gains for a lower ICER.

*BMI* Body mass index, *ICER* Incremental cost-effectiveness ratio, *N/A* Not applicable, *QALY* Quality adjusted life year, *RYGB* Roux-en-Y gastric bypass surgery, *WMP1* Weight Management Programme 1, *WMP2* Weight Management Programme 2, *VLCD* Very Low Calorie Diet.

RYGB was the most costly intervention, at a cost per patient of about £25,000 (undiscounted costs) or £20,000 (discounted costs), including intensive surgical preparation, surgery, and long-term follow-up with potential for costly complications and revision surgery. Whilst surgery is expensive to deliver, cost savings associated with reducing obesity related diseases offset about 30% of the intervention delivery cost. Surgery delivers superior weight loss, sustained over the longer term and thus leads to substantial QALY gains compared to all other interventions.

Compared with no population intervention (i.e. current BMI trends), WMPs and RYGB all have ICERs < £30,000 per QALY gained. Assuming a five-year weight regain, the Look AHEAD intervention has an ICER of £23,725 per QALY gained but this reduces to £14,906 if the longer term weight loss trends from the trial are extrapolated. A WMP, similar to that recommended by NICE for Tier 2 (i.e. WMP1), is comparatively cheap to deliver at scale, generates small QALY gains (~0.19 per person, discounted over 30 years) but has the lowest ICER costing an additional £557 per QALY gained compared to baseline.

For the fully incremental analysis under base case weight regain assumptions, VLCDs, WMP2 and Look AHEAD are excluded because they are either dominated (where an alternative intervention is less costly and more beneficial) or extendedly dominated interventions (where an alternative is more effective, but with a lower ICER) and cannot therefore be considered the most cost-effective option overall. Of the two remaining non-dominated alternatives (WMP1 and RYGB), RYGB is more costly, but also delivers greater QALY gains and has an ICER of £11,648 compared to WMP1.

Several additional specific comparisons between interventions are possible using the output from the micro-simulation model. First, adding a VLCD to WMP1 generates little incremental benefit for substantial extra cost, with an ICER of ~£121,000 per QALY gained. Sensitivity analyses show that applying a linear rather than five-year weight regain assumption favours Look AHEAD, but reduces the cost-effectiveness of VLCDs. Further sensitivity analyses around the model time horizon and discount rate show that lower discount rates favour surgery, whilst higher discount rates favour WMP1 (see Supplementary Material 3 for further details). Longer model time horizons increase the cost-effectiveness of more effective interventions as the full impact of obesity related disease on healthcare costs and patient quality and length of life are realised.

## Discussion

RYGB delivers substantial weight loss, generates large QALY gains and is the most cost-effective use of scarce NHS resources for the general population of adults with BMI ≥ 35 kg/m^2^ (assuming a willingness to pay of £20,000 per QALY). However, surgery is expensive (£8253 per case) and widespread upfront delivery of RYGB to all people with severe obesity in the UK adult population (12% of 50.8 million adults) would place massive strain on fixed NHS resources (an estimated cost of £5 billion at 10% uptake), even if it represents a cost-effective use of resources longer term. Moreover, many people will not opt for surgery and it is unlikely health services would be able to deliver so many surgeries, at least in the short term, due to operational delivery constraints, even if the budgets would be made available.

In scenarios where the NHS cannot afford or patient preference excludes bariatric surgery as an option, a short duration, low intensity behavioural WMP typically delivered in hour long group sessions over 12 weeks offering advice on diet and physical activity is a cost-effective use of resources that can likely be delivered at scale. However, we find that adding a VLCD to a diet and lifestyle intervention is not a cost-effective use of resources.

We find that the cost-effectiveness of the much more intensive Look AHEAD WMP intervention, for which excellent long-term data are available, is sensitive to assumptions about the rate of weight regain over time. Under a 5-year weight regain assumption, Look AHEAD is borderline cost-effective, but the ICER decreases when assuming a linear regain trend over time. The latter may be more appropriate and in line with recently published Look AHEAD data showing that in the 2 years after cessation of the intervention, there was no evidence of any weight regain, improving the case for cost-effectiveness further [30].

### Strengths and limitations

Strengths of this study are the systematic approach taken, and the use of the best available, nationally representative data. One distinctive advantage of our modelling approach is the potential to account for the risks, and utility decrements associated with multi-morbidity health states (e.g. diabetes and hypertension). We use best practice methods, using a multiplicative approach to assign utility values to multi-morbid health states [31]. The quality of the epidemiological and cost outputs is bolstered by the systematic search for evidence used by the UKHF microsimulation model [32].

There were several limitations. Disease costs only include direct costs and do not account for indirect healthcare costs or non-healthcare costs, such as loss of productivity, due to data availability. Additionally, the model does not include costs associated with a healthier, longer living population, through increased risk of other diseases of old age.

Several obesity related diseases, including gallstones, musculoskeletal conditions such as back pain, sleep apnoea, infertility, non-alcoholic fatty liver disease were not included due to a lack of available data. CHD was modelled using incidence of myocardial infarction (not angina and chronic heart failure). These omissions are major cost drivers and their exclusion likely underestimates disease burden and cost-effectiveness of weight loss interventions [32]. Furthermore, while the UKHF model can incorporate multi-stage diseases such as T2DM, this was not undertaken for this project due to resource and time limitations.

We have chosen to assign a utility value of 1 (equivalent to full health) to patients that do not have obesity related disease. This could be argued to overestimate the utility gains for a population who are not in full health, but have no obesity related disease. The alternative approach, assuming general population utility norms would be inappropriate, as the general population values already implicitly include the utility decrements associated with highly prevalent obesity related diseases such as diabetes, cancer, hypertension and CHD. Nonetheless, we acknowledge that the true utility for the absence of obesity related disease is unknown and is thus an area of uncertainty.

It was not possible to investigate the impact of parametric uncertainty, for example through probabilistic sensitivity analyses, on the model outputs such as estimates of the ICER. This means that our results will understate the uncertainty around the conclusions. This is a limitation common to many large micro-simulation models, such as the one used for this project. There are however, important advantages of using the microsimulation model that offset this limitation. For example, recent evidence from a comparison of diabetes’ models suggests that microsimulation models provide less biased estimates of the ICER, despite their lack of flexibility to explore issues such as parameter uncertainty [33]. Ideally, a model would be able to achieve both unbiased ICER estimation and provide a comprehensive exploration of parameter uncertainty. However, this would require undertaking many thousands of consecutive model runs, and would require a high performance computer. Unfortunately, it was beyond the resources and scope of the current project to do this. However, previous work undertaken using the UKHF microsimulation model has identified uncertainty surrounding BMI change data to be one of the most important drivers of quality adjusted life years when compared to the impact from relative risks of obesity related diseases, stroke and colorectal cancer [34].

The results are based on the assumption that one intervention only is given at the start of the 30-year period for all those with a BMI ≥ 35 kg/m^2^ in 2016, with no repetition or introduction of new interventions over time. This may be true for bariatric surgery, but individuals are likely to undertake several WMPs during their lifetime if they lose weight then regain it. The net impact of additional intervention cost and potential improvements in health outcomes on cost-effectiveness is unclear and the optimal sequencing of different interventions warrants further investigation.

Finally, modelling assumes that the effect on health depends on current BMI, not past BMI. This therefore assumes that the current in-year risk of incident disease for a cohort that has lost weight from 35 kg/m^2^ to 30 kg/m^2^ is the same as a cohort who have remained at 30 kg/m^2^. However, their lifetime incidence will be different because of the prior time at higher risk for the cohort with a starting BMI of 35 kg/m^2^ and the assumption that this cohort will return to that starting weight. These assumptions are not testable, but are consonant with epidemiological evidence that the risk of obesity depends upon the person-years of exposure to it and the biology of key obesity related diseases, hypertension, diabetes, and cardiovascular disease [35].

### Existing literature

The published literature provides variable quality cost-effectiveness evidence for weight loss interventions for adults with severe obesity in the UK. Some studies fail to extrapolate benefits over the longer-term, for example using decision modelling studies [36, 37] and thus may miss longer term implications of weight loss that cannot be captured directly within short term clinical trials. Five published decision analysis models of WMPs in the UK setting were identified with mixed results. A physical activity programme and dietary advice for men delivered in a football club setting was cost-effective compared to a weight loss booklet over a modelled lifetime horizon [38]. Referrals from health professionals to WMPs tended to be cost-effective compared to information or minimalist interventions [39–41]. The Counterweight programme delivered in UK primary care by a practice nurse was less costly and more effective than no treatment [41]. Overall, these findings are in agreement with our comparison of WMPs vs. general population obesity trends. An economic evaluation of the BWeL trial found that a brief opportunistic intervention that facilitates and supports referral to a behavioural WMP is cost-effective in those with a BMI > 30 kg/m^2^ [42].

We are aware of one UK study that found that a VLCD weight management programme that provided food packs, behaviour change therapy and group support (Lighter Life Total) was cost-effective compared to no treatment, Slimming World®, Counterweight and Weight Watchers® (with similar support) in those with BMI ≥ 30 kg/m^2^. However, the intervention was less effective than surgery for those with BMI ≥ 40 kg/m^2^ [43, 44]. The comparison with ‘do nothing’ indicates cost-effectiveness and generates a similar conclusion to our analysis. However, the study does not evaluate the incremental effect of adding a VLCD component to an existing WMP, compared with an existing WMP alone. Our study adds to the existing literature and finds that adding a VLCD to an existing WMP is not a cost-effective use of resources, and a simple WMP alone is likely to offer better value for money. Our study also evaluates different intensity WMPs against each other, the incremental benefit of VLCDs added to WMPs, and the comparative cost-effectiveness of these WMPs relative to surgery.

A recently published within trial cost-effectiveness analysis of the Look AHEAD intervention found that the intervention was more costly (+$6666) than standard diabetes support and education [15]. However, the cost-effectiveness case was less clear, and dependent on the measure of utilities used for the analysis.

Consistent with the existing literature, we find that surgery is cost-effective, but we find no evidence of long-term cost savings as noted in other studies. This is despite our analysis including a wide range of obesity related diseases. One reason is that, unlike many studies, our model included a wider range of pre and post-operative care costs, such as management of complications, and ongoing monitoring and review.

## Conclusions

For adults with severe obesity, RYGB surgery was the most expensive, but also the most beneficial intervention in terms of QALY gains and can thus be considered most efficient. Lifestyle WMPs are likely to be cost-effective compared with no intervention, and adding a VLCD to a WMP was not found to be cost-effective.

## Disclaimer

The views and opinions expressed therein are those of the authors and do not necessarily reflect those of the Department of Health, or the funders that provide institutional support for the authors of this study.

## Supplementary information

Technical appendix

Intervention costs

Sensitivity analyses

## Data Availability

All data relevant to the study are included in the article or available here https://www.journalslibrary.nihr.ac.uk/programmes/hta/150904/#/
