# Correction: Cost-effectiveness of bariatric surgery and non-surgical weight management programmes for adults with severe obesity: a decision analysis model

**DOI:** 10.1038/s41366-021-00931-1

**Published:** 2021-08-26

**Authors:** D. Boyers, L. Retat, E. Jacobsen, A. Avenell, P. Aveyard, E. Corbould, A. Jaccard, D. Cooper, C. Robertson, M. Aceves-Martins, B. Xu, Z. Skea, M. de Bruin, and the REBALANCE team, Elisabet Jacobsen, Dwayne Boyers, David Cooper, Lise Retat, Paul Aveyard, Fiona Stewart, Graeme MacLennan, Laura Webber, Emily Corbould, Benshuai Xu, Abbygail Jaccard, Bonnie Boyle, Eilidh Duncan, Michal Shimonovich, Cynthia Fraser, Lara Kemp

**Affiliations:** 1 Health Economics Research Unit, University of Aberdeen, Aberdeen, UK; 2 UK Health Forum, London, UK; 3 Health Services Research Unit, University of Aberdeen, Aberdeen, UK; 4 Nuffield Department of Primary Care Health Sciences, Oxford University, Oxford, UK; 5 NIHR Oxford Biomedical Research Centre (BRC) Obesity, Diet and Lifestyle Theme, Oxford, UK; 6 NIHR Applied Research Collaboration (ARC) Oxford and Thames Valley, Oxford, UK; 7 Health Psychology, University of Aberdeen, Aberdeen, UK; 8 Radboud University Medical Center, IQ Healthcare, Radboud Institute for Health Sciences, Nijmegen, The Netherlands

**Keywords:** Weight management, Lifestyle modification, Health policy

Correction to: *International Journal of Obesity* 10.1038/s41366-021-00849-8

The original version of this article unfortunately omitted two affiliations for Professor Paul Aveyard—NIHR Oxford Biomedical Research Centre (BRC) Obesity, Diet and Lifestyle Theme and NIHR Applied Research Collaboration (ARC) Oxford and Thames Valley. In addition, it was noted some affiliations showed duplications where authors have been listed by both full name and initial/surname. We apologise for this error. The original article has been corrected.

