# Supplementary material for: Cost-effectiveness of bariatric surgery and non-surgical weight management programmes for adults with severe obesity: a decision analysis model
Source: Int J Obes (Lond). 2021 Jun 4;45(10):2179–90. doi: 10.1038/s41366-021-00849-8 (PMC8455321; doi:10.1038/s41366-021-00849-8)
Supplement: Supplementary file 1 — Technical appendix [file 41366_2021_849_MOESM1_ESM.pdf]

Technical Appendix

Contents

Data inputs 3

UKHF microsimulation methodology 41

UKHF tool methodology **Error! Bookmark not defined.**

References 56

Data inputs

Epidemiological data

Population data

Demographic data was collected for England, Scotland, Wales and Northern Ireland. Information was collected on the age and sex distribution of the population, and the distribution of deaths by age and sex.

The data were processed as text files, in a format suitable for inclusion in the microsimulation programme. The data sources were as follows

Table 1: Population data sources by geography

Demography	Geography	Source
Total population by age and sex	UK	ONS. Population Estimates for UK, England and Wales, Scotland and Northern Ireland: mid-2015. 2016.(1)
Deaths by age and sex	UK	ONS. Deaths registed in Engalnd and Wales 2015. 2016 (2)<br>National Records of Scotland. Deaths, by sex and single year of age, Scotland 1974 to 2016. 2017 (3)<br>NISRA. Deaths by single year of age, 1955 to 2015, 2017(4)

Disease data

A number of obesity-related diseases were modelled (see Table 2). The list of diseases modelled for obesity was determined after a review of the literature conducted for the WRAP study (5)(Table 2).

Table 2: Characteristics of diseases modelled

	Duration	Terminal	Age category
Cardiovascular outcomes			
CHD	Chronic	Yes	Adult
Diabetes Mellitus (Type 2)	Chronic	No	Adult
Hypertension	Chronic	No	Adult
Stroke	Chronic	Yes	Adult
Cancer and other outcomes			

Breast cancer	Chronic	Yes	Adult (post menopausal women only)
Colorectal cancer	Chronic	Yes	Adult
Endometrial cancer	Chronic	Yes	Adult (Female only)
Knee Osetoarthritis	Chronic	No	Adult
Kidney cancer	Chronic	Yes	Adult
Lung cancer	Chronic	Yes	Adult
Oesphoageal cancer	Chronic	Yes	Adult
Ovarian cancer	Chronic	Yes	Adult (Female only)
Pancreatic cancer	Chronic	Yes	Adult

All diseases were lifelong, chronic diseases, so once acquired, were prevalent for the duration of an individual's life. Individuals could develop more than one diseases, but these were considered independent of one another. All diseases apart from diabetes, hypertension and knee osteoathritis were terminal. Epidemiological data on each disease's incidence, prevalence, mortality and survival was collected (see Table 3). When a parameter, e.g. Survival was not available from the literature or national statistics, this was computed – see Module two: Microsimulation model section Approximating missing disease statistics for methods.

Summary of data sources

Table 3: Summary of disease data sources

Diseases	Incidence	Prevalence	Mortality	Survival	Relative Risk
Breast cancer	ONS, Cancer registration statistics, 2015	NA	ONS, Cancer registration statistics, 2015	ONS, Cancer survival in England: Patients diagnosed between 2010 and 2014 and followed up to 2015	World Obesity Federation (DYNAMO project)
CHD	Computed from Prevalence and Mortality	BHF, Cardiovascular Disease Statistics 2014 (6)	CVD statistics, 2017	Computed from prevalence and mortality	World Obesity Federation (DYNAMO project)
Colorectal cancer	ONS, Cancer registration statistics, 2015	NA	ONS, Cancer registration statistics, 2015	ONS, Cancer survival in England: Patients diagnosed between 2010 and 2014 and followed up to 2015	World Obesity Federation (DYNAMO project)
Diabetes	Personal communication with Dr Craig Curry from Cardiff University	International Diabetes Federation, 2012	Non-terminal	Non-terminal	Derived from PREVEND data (Jaccard 2015 et al.)
Endometrial cancer	ONS, Cancer registration statistics, 2015	NA	ONS, Cancer registration statistics, 2015	ONS, Cancer survival in England: Patients diagnosed between	World Obesity Federation (DYNAMO project)

				2010 and 2014 and followed up to 2015	
Hypertension	Derived from Prevalence	Health survey for England, 2015	Non-terminal	Non-terminal	World Obesity Federation (DYNAMO project)
Knee Osetoarthrits	Derived from prevalence	Arthritis UK Musculoskeletal calculator (7)	Non-terminal	Non-terminal	Zheng et al (2015) (8)
Oesophageal cancer	ONS, Cancer registration statistics, 2015	NA	ONS, Cancer registration statistics, 2015	ONS, Cancer survival in England: Patients diagnosed between 2010 and 2014 and followed up to 2015	World Obesity Federation (DYNAMO project)
Ovarian cancer	ONS, Cancer registration statistics, 2015	NA	ONS, Cancer registration statistics, 2015	ONS, Cancer survival in England: Patients diagnosed between 2010 and 2014 and followed up to 2015	World Obesity Federation (DYNAMO project)
Pancreatic cancer	ONS, Cancer registration statistics, 2015	NA	ONS, Cancer registration statistics, 2015	ONS, Cancer survival in England: Patients diagnosed between 2010 and 2014 and followed up to 2015	World Obesity Federation (DYNAMO project)
Kindey cancer	ONS, Cancer registration statistics, 2015	NA	ONS, Cancer registration statistics, 2015	ONS, Cancer survival in England: Patients diagnosed between	World Obesity Federation (DYNAMO project)

				2010 and 2014 and followed up to 2015	
Stroke	BHF, stroke statistics 2009 (9)	BHF, Cardiovascular Disease Statistics 2014 (6)	ONS, Deaths Registrations Summary Statistics, England and Wales, 2015 (2)	Computed from prevalence and mortality	World Obesity Federation (DYNAMO project) (10)

Incidence, Prevalence, Mortality data by disease

Breast cancer

Prevalence data was not available on breast cancer data, but the model does not require the input of prevalence, only of incidence, so this parameter was not required.

Table 4: Breast cancer epidemiological data (per 100,000 population)

Incidence			Prevalence			Mortality		
ONS, Cancer registration statistics, 2015(11)			Prevalence is not a required input into the model			ONS, Cancer registration statistics, 2015(11)		
ICD 10: C50			N/A			ICD 10: C50		
Age group	Male	Female	Age group	Male	Female	Age group	Male	Female
0-49	N/A	N/A	N/A	N/A	N/A	0-49	N/A	N/A
50-54	N/A	279.4	N/A	N/A	N/A	50-54	N/A	35.7
55-59	N/A	277.0	N/A	N/A	N/A	55-59	N/A	42.0
60-64	N/A	342.2	N/A	N/A	N/A	60-64	N/A	48.4
65-69	N/A	418.8	N/A	N/A	N/A	65-69	N/A	60.7
70-74	N/A	373.6	N/A	N/A	N/A	70-74	N/A	76.6
75-79	N/A	399.6	N/A	N/A	N/A	75-79	N/A	119.3
80-84	N/A	453.0	N/A	N/A	N/A	80-84	N/A	166.4
85-89	N/A	476.0	N/A	N/A	N/A	85-89	N/A	224.4
90+	N/A	451.0	N/A	N/A	N/A	90+	N/A	340.4

Coronary heart disease (CHD)

Table 5: CHD epidemiological data (per 100,000 population)

Incidence			Prevalence			Mortality		
Smolina et al. 2012(12)			BHF CVD Stats 2014(6)			ONS 2015(2)		
ICD 10: I21-I22			ICD 10: I21			ICD 10: I21-I22		
Age group	Male	Female	Age group	Male	Female	Age group	Male	Female
0-29	0.0	0.0	0-44	60.0	30.0	<1	0.0	0.3
30-54	88.1	21.2	45-54	1070.0	430.0	1-4	0.0	0.0
55-64	317.0	90.3	55-64	4510.0	1240.0	5-9	0.0	0.0
65-74	533.0	237.0	65-74	8660.0	2960.0	15-24	0.1	0.0
75-84	1017.0	597.0	75+	14780.0	6960.0	25-34	0.8	0.2
85+	1987.0	1395.0				35-44	4.9	1.4
						45-54	21.2	5.2
						55-64	52.2	14.2
						65-74	109.4	44.6
						75-84	281.0	146.0

						85+	692.0	454.7

Colorectal cancer

Prevalence data was not available on colorectal cancer data, but the model does not require the input of prevalence, only of incidence, so this parameter was not required.

Table 6: Colorectal cancer epidemiological data (per 100,000 population)

**Incidence**			**Prevalence**			**Mortality**		
ONS, Cancer registration statistics, 2015(11)			Prevalence is not a required input into the model			ONS, Cancer registration statistics, 2015(11)		
ICD 10: C18-C20			N/A			ICD 10: C18-C20		
Age group	Male	Female	Age group	Male	Female	Age group	Male	Female
0-9	0.0	0.0	N/A	N/A	N/A	0-19	0.0	0.0
10-14	0.3	0.8	N/A	N/A	N/A	20-24	0.3	0.8
15-19	1.6	2.2	N/A	N/A	N/A	25-29	0.4	0.4
20-24	2.3	3.1	N/A	N/A	N/A	30-34	1.5	1.2
25-29	2.3	2.7	N/A	N/A	N/A	35-39	2.5	2.5
30-34	5.6	6.5	N/A	N/A	N/A	40-44	4.0	2.7
35-39	9.1	10.7	N/A	N/A	N/A	45-49	5.8	5.6
40-44	12.0	11.8	N/A	N/A	N/A	50-54	12.7	10.2
45-49	23.2	21.5	N/A	N/A	N/A	55-59	24.3	15.3
50-54	42.6	37.6	N/A	N/A	N/A	60-64	40.3	23.7
55-59	84.2	61.8	N/A	N/A	N/A	65-69	57.6	34.2
60-64	150.3	91.4	N/A	N/A	N/A	70-74	87.9	53.7
65-69	196.1	118.2	N/A	N/A	N/A	75-79	131.8	80.4
70-74	276.8	172.1	N/A	N/A	N/A	80-84	213.3	139.8
75-79	373.8	235.6	N/A	N/A	N/A	85-89	313.4	215.0
80-84	457.5	309.3	N/A	N/A	N/A	90+	410.4	264.8
85-89	511.9	359.5	N/A	N/A	N/A			
90+	460.3	304.2	N/A	N/A	N/A			

Diabetes Type 2

Table 7: Diabetes type 2 incidence and prevalence estimates (per 100,000 population)

**Incidence**			**Prevalence**			**Mortality**
Personal communication with Dr Curry from Cardiff University (13)			National Diabetes Audit 2015-2016(14)			Non-terminal
ICD 10 codes unknown			ICD 10 codes unknown			
Age group	Male	Female	Age group	Male	Female	
0-4	56	53	0-4	1.999	2.727	
5-9	34	42	5-9	6.681	6.372	
10-14	43	40	10-14	15	19.285	
15-19	83	107	15-19	41.744	64.613	
20-24	75	145	20-24	85.329	160.621	
25-29	101	226	25-29	202.748	352.739	
30-34	150	242	30-34	561.584	684.461	
35-39	240	263	35-39	1361.296	1249.819	
40-44	355	333	40-44	2617.251	1898.323	
45-49	561	482	45-49	4338.317	2858.298	
50-54	820	636	50-54	6451.945	4227.206	
55-59	1068	847	55-59	9371.893	6188.7	
60-64	1316	965	60-64	11825.85	7780.135	
65-69	1516	1234	65-69	13621.13	9047.041	
70-74	1763	1378	70-74	16010.86	11196.63	
75-79	1677	1483	75-79	18065.24	13559.67	
80-84	1645	1336	80-84	18464.43	14217.44	
85-89	1300	1169	85+	15210.91	11513.66	
90+	546	440				

Endometrial cancer

Prevalence data was not available on endometrial cancer data, but the model does not require the input of prevalence, only of incidence, so this parameter was not required.

Table 8: Endometrial cancer epidemiological data (per 100,000 population)

**Incidence**			**Prevalence**			**Mortality**		
ONS, Cancer registration statistics, 2015(11)			Prevalence is not a required input into the model			ONS, Cancer registration statistics, 2015(11)		
ICD 10: C33-C34			N/A			ICD 10: C33-C34		
Age group	Male	Female	Age group	Male	Female	Age group	Male	Female
0-24	N/A	0.0	N/A	N/A	N/A	0-29	N/A	0.0
25-29	N/A	0.8	N/A	N/A	N/A	30-34	N/A	0.1
30-34	N/A	1.3	N/A	N/A	N/A	35-39	N/A	0.2
35-39	N/A	2.2	N/A	N/A	N/A	40-44	N/A	0.7
40-44	N/A	6.5	N/A	N/A	N/A	45-49	N/A	0.8
45-49	N/A	11.7	N/A	N/A	N/A	50-54	N/A	1.1
50-54	N/A	30.4	N/A	N/A	N/A	55-59	N/A	4.5
55-59	N/A	52.0	N/A	N/A	N/A	60-64	N/A	8.9
60-64	N/A	67.9	N/A	N/A	N/A	65-69	N/A	14.4
65-69	N/A	82.7	N/A	N/A	N/A	70-74	N/A	19.2
70-74	N/A	83.3	N/A	N/A	N/A	75-79	N/A	27.3
75-79	N/A	90.6	N/A	N/A	N/A	80-84	N/A	32.8
80-84	N/A	82.5	N/A	N/A	N/A	85+	N/A	33.2
85-89	N/A	63.5	N/A	N/A	N/A			
90+	N/A	34.4	N/A	N/A	N/A			

Hypertension

Table 9: Hypertension incidence and prevalence estimates (per 100,000 population)

Incidence			Prevalence			Mortality
Derived from Prevalence			Health survey for England, 2015(15)			Non-terminal
ICD 10 codes unknown			Defined in this survey as SBP at least 140mmHg or DBP at least 90mmHg or on medication prescribed to control hypertension			
Age group	Male	Female	Age group	Male	Female	
0-17	0.9	3.8	0-15	0.0	0.0	
18-29	98.1	9.0	16-24	490.0	130.0	
30-39	62.2	73.7	25-34	1090.0	150.0	
40-49	140.4	114.3	35-44	1800.0	910.0	
50-59	274.2	360.0	45-54	3170.0	2050.0	
60-110	10.3	42.7	55-64	5000.0	3990.0	
			65-74	5870.0	5830.0	
			75-110	6160.0	7030.0	

Knee Osteoarthritis

Table 10: Knee Osteoarthritis incidence and prevalence estimates (per 100,000 population)

**Incidence**			**Prevalence**			**Mortality**
Derived from prevalence			Arthritis UK, 2016(7)			Non-terminal
ICD 10 codes unknown			ICD 10 codes unknown			
Age group	Male	Female	Age group	Male	Female	
0-17	0.0	0.0	0-44	0.0	0.0	
18-29	0.0	0.0	45-64	169.5	202.0	
30-39	14.0	0.4	65-74	173.4	209.4	
40-49	0.0	17.1	75+	139.7	170.4	
50-59	0.0	0.0				
60-79	0.0	0.0				
80+	0.0	0.0				

Renal cancer

Prevalence data was not available on Renal cancer data, but the model does not require the input of prevalence, only of incidence, so this parameter was not required.

Table 4: Renal cancer epidemiological data (per 100,000 population)

**Incidence**			**Prevalence**			**Mortality**		
ONS, Cancer registration statistics, 2015(11)			Prevalence is not a required input into the model			ONS, Cancer registration statistics, 2015(11)		
ICD 10: C64-C68			N/A			ICD 10: C64-C68		
Age group	Male	Female	Age group	Male	Female	Age group	Male	Female
<1	2.1	1.9	N/A	N/A	N/A	<1	0.0	0.0
1-4	1.8	2.5	N/A	N/A	N/A	1-4	0.0	0.2
5-9	0.5	0.7	N/A	N/A	N/A	5-9	0.0	0.0
10-19	0.0	0.0	N/A	N/A	N/A	10-19	0.0	0.0
20-24	0.2	0.4	N/A	N/A	N/A	20-24	0.0	0.0
25-29	0.7	1.0	N/A	N/A	N/A	25-29	0.0	0.0
30-34	1.3	0.8	N/A	N/A	N/A	30-34	0.0	0.0
35-39	4.4	2.2	N/A	N/A	N/A	35-39	0.6	0.3
40-44	0.9	4.3	N/A	N/A	N/A	40-44	0.8	0.5
45-49	16.5	8.5	N/A	N/A	N/A	45-49	3.4	1.7
50-54	23.9	11.4	N/A	N/A	N/A	50-54	4.4	1.8
55-59	37.7	17.9	N/A	N/A	N/A	55-59	9.8	3.1
60-64	55.0	23.6	N/A	N/A	N/A	60-64	14.4	5.2
65-69	71.4	37.2	N/A	N/A	N/A	65-69	23.5	11.2
70-74	89.1	42.7	N/A	N/A	N/A	70-74	28.9	13.6
75-79	107.7	59.6	N/A	N/A	N/A	75-79	45.6	23.2
80-84	118.1	60.7	N/A	N/A	N/A	80-84	58.9	31.6
85-89	129.6	65.5	N/A	N/A	N/A	85-89	81.6	37.9
90+	104.0	514.0	N/A	N/A	N/A	90+	96.3	44.9

Oesophageal cancer

Prevalence data was not available on Oesophageal cancer data, but the model does not require the input of prevalence, only of incidence, so this parameter was not required.

Table 12: Oesophageal cancer incidence and prevalence estimates (per 100,000 population)

**Incidence**			**Prevalence**			**Mortality**		
ONS, Cancer registration statistics, 2015(11)			Prevalence is not a required input into the model			ONS, Cancer registration statistics, 2015(11)		
ICD 10 C15			N/A			ICD 10 C15		
Age group	Male	Female	Age group	Male	Female	Age group	Male	Female
0-4	0.0	0.0	N/A	N/A	N/A	0-4	0.0	0.0
5-9	0.0	0.0	N/A	N/A	N/A	5-9	0.0	0.0
10-14	0.0	0.0	N/A	N/A	N/A	10-14	0.0	0.0
15-19	0.0	0.0	N/A	N/A	N/A	15-19	0.0	0.0
20-24	0.0	0.0	N/A	N/A	N/A	20-24	0.0	0.0
25-29	0.2	0.0	N/A	N/A	N/A	25-29	0.0	0.0
30-34	0.4	0.0	N/A	N/A	N/A	30-34	0.3	0.0
35-39	1.2	0.3	N/A	N/A	N/A	35-39	0.7	0.2
40-44	2.2	1.1	N/A	N/A	N/A	40-44	2.0	0.7
45-49	5.4	1.6	N/A	N/A	N/A	45-49	4.2	1.1
50-54	12.7	3.9	N/A	N/A	N/A	50-54	7.8	2.5
55-59	29	9.1	N/A	N/A	N/A	55-59	21.3	4.8
60-64	44.8	13.3	N/A	N/A	N/A	60-64	33.9	10.1
65-69	60.8	31.1	N/A	N/A	N/A	65-69	51.6	12.6
70-74	86.7	27.6	N/A	N/A	N/A	70-74	72.6	22.1
75-79	89.2	36.7	N/A	N/A	N/A	75-79	77.6	32.4
80-84	106.9	51	N/A	N/A	N/A	80-84	106.4	46.2
85-89	114.9	59.3	N/A	N/A	N/A	85-89	119.7	69.3
90+	101.9	66.3	N/A	N/A	N/A	90+	135.4	68.1

Ovarian cancer

Prevalence data was not available on Ovarian cancer data, but the model does not require the input of prevalence, only of incidence, so this parameter was not required.

Table 13: Ovarian cancer incidence and prevalence estimates (per 100,000 population)

**Incidence**			**Prevalence**			**Mortality**		
ONS, Cancer registration statistics, 2015(11)			Prevalence is not a required input into the model			ONS, Cancer registration statistics, 2015(11)		
ICD 10 C56						ICD 10 C56		
Age group	Male	Female	Age group	Male	Female	Age group	Male	Female
0-4	N/A	0.0	N/A	N/A	N/A	0-19	N/A	0.0
5-9	N/A	0.2	N/A	N/A	N/A	20-29	N/A	0.3
10-14	N/A	0.6	N/A	N/A	N/A	30-34	N/A	0.5
15-19	N/A	1.6	N/A	N/A	N/A	35-39	N/A	1.0
20-24	N/A	4.0	N/A	N/A	N/A	40-44	N/A	2.1
25-29	N/A	5.0	N/A	N/A	N/A	45-49	N/A	3.9
30-34	N/A	5.5	N/A	N/A	N/A	50-54	N/A	8.1
35-39	N/A	8.2	N/A	N/A	N/A	55-59	N/A	12.6
40-44	N/A	13.6	N/A	N/A	N/A	60-64	N/A	20.6
45-49	N/A	19.9	N/A	N/A	N/A	65-69	N/A	33.8
50-54	N/A	28.1	N/A	N/A	N/A	70-74	N/A	43.2
55-59	N/A	36.6	N/A	N/A	N/A	75-79	N/A	58.4
60-64	N/A	40.9	N/A	N/A	N/A	80-84	N/A	62.5
65-69	N/A	56.5	N/A	N/A	N/A	85-89	N/A	71.1
70-74	N/A	61.2	N/A	N/A	N/A	90+	N/A	59.5
75-79	N/A	71.0	N/A	N/A	N/A			
80-84	N/A	70.6	N/A	N/A	N/A			
85-89	N/A	69.1	N/A	N/A	N/A			
90+	N/A	53.8	N/A	N/A	N/A			

Pancreatic cancer

Prevalence data was not available on Pancreatic cancer data, but the model does not require the input of prevalence, only of incidence, so this parameter was not required.

Table 14: Pancreatic cancer incidence and prevalence estimates (per 100,000 population)

**Incidence**			**Prevalence**			**Mortality**		
ONS, Cancer registration statistics, 2015(11)			Prevalence is not a required input into the model			ONS, Cancer registration statistics, 2015(11)		
ICD 10 C25						ICD 10 C25		
Age group	Male	Female	Age group	Male	Female	Age group	Male	Female
0-19	0.0	0.0	N/A	N/A	N/A	0-34	0.0	0.0
20-24	0.0	0.2	N/A	N/A	N/A	35-39	0.3	0.2
25-29	0.2	0.3	N/A	N/A	N/A	40-44	1.6	1.3
30-34	0.5	0.7	N/A	N/A	N/A	45-49	3.3	2.8
35-39	1.0	0.7	N/A	N/A	N/A	50-54	8.4	6.1
40-44	2.8	1.9	N/A	N/A	N/A	55-59	15.2	10.6
45-49	4.1	3.8	N/A	N/A	N/A	60-64	25.4	21.3
50-54	10.5	7.9	N/A	N/A	N/A	65-69	39.9	29.1
55-59	17.5	12.9	N/A	N/A	N/A	70-74	57.2	43.8
60-64	29.7	23.1	N/A	N/A	N/A	75-79	75.8	61.5
65-69	47.0	32.3	N/A	N/A	N/A	80-84	99.9	82.0
70-74	62.7	48.3	N/A	N/A	N/A	85-89	109.2	95.1
75-79	82.8	71.4	N/A	N/A	N/A	90+	106.9	109.4
80-84	99.4	88.9	N/A	N/A	N/A			
85-89	111.1	101.8	N/A	N/A	N/A			
90+	101.2	99.5	N/A	N/A	N/A			

Stroke

Table 15: Stroke epidemiological data (per 100,000 population)

**Incidence**			**Prevalence**			**Mortality**		
Computed from Prevalence and Mortality			BHF CVD Stats 2014(6)			ONS 2015(2)		
Based on general practice records, ICD codes not given			ICD 10: I60-I69			ICD: I60-I64		
Age group	Male	Female	Age group	Male	Female	Age group	Male	Female
0-44	0.0	0.0	0-44	100.0	110.0	<1	0.0	0.0
45-54	273.8.	249.3	45-54	850.0	750.0	1-4	0.0	0.0
55-64	610.3	324.1	55-64	2600.0	1800.0	5-14	0.0	0.0
65-74	1314.6	1024.2	65-74	6080.0	4160.0	15-24	0.2	0.0
75+	2906.2	2566.2	75+	14550.0	12170.0	25-34	0.1	0.1
						35-44	0.7	0.5
						45-54	3.3	2.3
						55-64	18.6	9.1
						65-74	67.1	45.8
						75-84	359.7	288.3
						85+	1335.1	1480.2

Survival data

Survival statistics for CHD and Stroke were not identified in the literature. We modelled these using prevalence and mortality data, – see *Module two: Microsimulation model* section *Approximating missing disease statistics* for methods.

CHD

Table 16: Probability of 1, 5 and 10 year survival computed from prevalence and mortality data for Coronary Heart Disease.

	**Survival probability – 1 year**		**Survival probability – 5 year**		**Survival probability – 10 year**	
Age	M	F	M	F	M	F
1-5	1.000	1.000	1.000	1.000	1.000	1.000
6	0.500	1.000	0.500	1.000	0.500	1.000
7	0.667	1.000	0.667	1.000	0.667	1.000
8	0.750	1.000	0.750	1.000	0.750	1.000
9	0.800	1.000	0.800	1.000	0.800	1.000
10	0.833	1.000	0.833	1.000	0.833	1.000
11	0.857	1.000	0.857	1.000	0.857	1.000
12	0.875	1.000	0.875	1.000	0.875	1.000
13	0.889	1.000	0.889	1.000	0.889	1.000
14	0.900	1.000	0.900	1.000	0.900	1.000
15	1.000	0.000	1.000	0.000	1.000	0.000
16	1.000	0.500	1.000	0.500	1.000	0.500
17	1.000	0.667	1.000	0.667	1.000	0.667
18	1.000	0.750	1.000	0.750	1.000	0.750
19	1.000	0.800	1.000	0.800	1.000	0.800
20	1.000	0.833	1.000	0.833	1.000	0.833
21	1.000	0.857	1.000	0.857	1.000	0.857
22	1.000	0.875	1.000	0.875	1.000	0.875
23	1.000	0.889	1.000	0.889	1.000	0.889
24	1.000	0.900	1.000	0.900	1.000	0.900
25	0.792	0.849	0.792	0.849	0.792	0.849
26	0.828	0.868	0.828	0.868	0.828	0.868
27	0.853	0.884	0.853	0.884	0.853	0.884
28	0.872	0.896	0.872	0.896	0.872	0.896

29	0.887	0.906	0.887	0.906	0.887	0.906
30	0.898	0.914	0.898	0.914	0.898	0.914
31	0.908	0.921	0.908	0.921	0.908	0.921
32	0.915	0.927	0.915	0.927	0.915	0.927
33	0.922	0.932	0.922	0.932	0.922	0.932
34	0.928	0.936	0.928	0.936	0.928	0.936
35	0.507	0.595	0.507	0.595	0.507	0.595
36	0.670	0.712	0.670	0.712	0.670	0.712
37	0.752	0.776	0.752	0.776	0.752	0.776
38	0.801	0.817	0.801	0.817	0.801	0.817
39	0.834	0.845	0.834	0.845	0.834	0.845
40	0.858	0.866	0.858	0.866	0.858	0.866
41	0.875	0.882	0.875	0.882	0.875	0.882
42	0.889	0.894	0.889	0.894	0.889	0.894
43	0.900	0.905	0.900	0.905	0.900	0.905
44	0.909	0.913	0.909	0.913	0.909	0.913
45	0.637	0.583	0.637	0.583	0.637	0.583
46	0.734	0.706	0.734	0.706	0.734	0.706
47	0.790	0.773	0.790	0.773	0.790	0.773
48	0.826	0.815	0.826	0.815	0.826	0.815
49	0.852	0.844	0.852	0.844	0.852	0.844
50	0.871	0.865	0.871	0.865	0.871	0.865
51	0.886	0.881	0.886	0.881	0.886	0.881
52	0.898	0.894	0.898	0.894	0.898	0.894
53	0.907	0.904	0.907	0.904	0.907	0.904
54	0.915	0.912	0.915	0.912	0.915	0.912
55	0.702	0.638	0.702	0.638	0.702	0.638
56	0.771	0.734	0.771	0.734	0.771	0.734
57	0.813	0.790	0.813	0.790	0.813	0.790
58	0.843	0.827	0.843	0.827	0.843	0.827
59	0.864	0.852	0.864	0.852	0.864	0.852
60	0.880	0.871	0.880	0.871	0.880	0.871
61	0.893	0.886	0.893	0.886	0.893	0.886
62	0.903	0.898	0.903	0.898	0.903	0.898
63	0.912	0.907	0.912	0.907	0.912	0.907
64	0.919	0.915	0.919	0.915	0.919	0.915

65	0.757	0.766	0.757	0.766	0.757	0.766
66	0.804	0.810	0.804	0.810	0.804	0.810
67	0.836	0.840	0.836	0.840	0.836	0.840
68	0.859	0.862	0.859	0.862	0.859	0.862
69	0.876	0.879	0.876	0.879	0.876	0.879
70	0.890	0.892	0.890	0.892	0.890	0.892
71	0.901	0.902	0.901	0.902	0.901	0.902
72	0.910	0.911	0.910	0.911	0.910	0.911
73	0.917	0.918	0.917	0.918	0.917	0.918
74	0.923	0.924	0.923	0.924	0.923	0.924
75	0.817	0.825	0.817	0.825	0.817	0.825
76	0.845	0.851	0.845	0.851	0.845	0.851
77	0.865	0.870	0.865	0.870	0.865	0.870
78	0.881	0.884	0.881	0.884	0.881	0.884
79	0.893	0.896	0.893	0.896	0.893	0.896
80	0.903	0.906	0.903	0.906	0.903	0.906
81	0.911	0.914	0.911	0.914	0.911	0.914
82	0.918	0.920	0.918	0.920	0.918	0.920
83	0.924	0.926	0.924	0.926	0.924	0.926
84	0.929	0.931	0.929	0.931	0.929	0.931
85	0.846	0.879	0.846	0.879	0.846	0.879
86	0.865	0.892	0.865	0.892	0.865	0.892
87	0.880	0.902	0.880	0.902	0.880	0.902
88	0.892	0.910	0.892	0.910	0.892	0.910
89	0.902	0.917	0.902	0.917	0.902	0.917
90	0.910	0.923	0.910	0.923	0.910	0.923
91	0.917	0.928	0.917	0.928	0.917	0.928
92	0.922	0.933	0.922	0.933	0.922	0.933
93	0.927	0.937	0.927	0.937	0.927	0.937
94	0.932	0.940	0.932	0.940	0.932	0.940
95	0.936	0.943	0.936	0.943	0.936	0.943
96	0.939	0.946	0.939	0.946	0.939	0.946
97	0.942	0.948	0.942	0.948	0.942	0.948
98	0.945	0.951	0.945	0.951	0.945	0.951
99	0.947	0.953	0.947	0.953	0.947	0.953
100	0.949	0.955	0.949	0.955	0.949	0.955

101	0.951	0.956	0.951	0.956	0.951	0.956
102	0.953	0.958	0.953	0.958	0.953	0.958
103	0.955	0.960	0.955	0.960	0.955	0.960
104	0.956	0.961	0.956	0.961	0.956	0.961
105	0.958	0.962	0.958	0.962	0.958	0.962
106	0.959	0.963	0.959	0.963	0.959	0.963
107	0.960	0.964	0.960	0.964	0.960	0.964
108	0.962	0.965	0.962	0.965	0.962	0.965
109	0.963	0.966	0.963	0.966	0.963	0.966
109+	0.963	0.966	0.963	0.966	0.963	0.966

Stroke

Table 17. Probability of 1, 5 and 10 year survival computed from prevalence and mortality data for Stroke.

Age	Stroke					
	Survival probability – 1 year		Survival probability – 5 year		Survival probability – 10 year	
	M	F	M	F	M	F
1	1.000	0.988	1.000	0.988	1.000	0.988
2	1.000	0.994	1.000	0.994	1.000	0.994
3	1.000	0.996	1.000	0.996	1.000	0.996
4	1.000	0.997	1.000	0.997	1.000	0.997
5	0.998	0.995	0.998	0.995	0.998	0.995
6	0.998	0.996	0.998	0.996	0.998	0.996
7	0.998	0.996	0.998	0.996	0.998	0.996
8	0.998	0.997	0.998	0.997	0.998	0.997
9	0.999	0.997	0.999	0.997	0.999	0.997
10	0.999	0.997	0.999	0.997	0.999	0.997
11	0.999	0.998	0.999	0.998	0.999	0.998
12	0.999	0.998	0.999	0.998	0.999	0.998
13	0.999	0.998	0.999	0.998	0.999	0.998
14	0.999	0.998	0.999	0.998	0.999	0.998
15	0.995	0.997	0.995	0.997	0.995	0.997
16	0.995	0.997	0.995	0.997	0.995	0.997
17	0.996	0.997	0.996	0.997	0.996	0.997
18	0.996	0.997	0.996	0.997	0.996	0.997
19	0.996	0.997	0.996	0.997	0.996	0.997
20	0.996	0.997	0.996	0.997	0.996	0.997
21	0.997	0.998	0.997	0.998	0.997	0.998
22	0.997	0.998	0.997	0.998	0.997	0.998
23	0.997	0.998	0.997	0.998	0.997	0.998
24	0.997	0.998	0.997	0.998	0.997	0.998
25	0.948	0.994	0.948	0.994	0.948	0.994
26	0.950	0.994	0.950	0.994	0.950	0.994
27	0.952	0.994	0.952	0.994	0.952	0.994
28	0.953	0.994	0.953	0.994	0.953	0.994
29	0.955	0.994	0.955	0.994	0.955	0.994

30	0.956	0.995	0.956	0.995	0.956	0.995
31	0.958	0.995	0.958	0.995	0.958	0.995
32	0.959	0.995	0.959	0.995	0.959	0.995
33	0.960	0.995	0.960	0.995	0.960	0.995
34	0.962	0.995	0.962	0.995	0.962	0.995
35	0.987	0.986	0.987	0.986	0.987	0.986
36	0.987	0.987	0.987	0.987	0.987	0.987
37	0.987	0.987	0.987	0.987	0.987	0.987
38	0.988	0.987	0.988	0.987	0.988	0.987
39	0.988	0.988	0.988	0.988	0.988	0.988
40	0.988	0.988	0.988	0.988	0.988	0.988
41	0.989	0.988	0.989	0.988	0.989	0.988
42	0.989	0.989	0.989	0.989	0.989	0.989
43	0.989	0.989	0.989	0.989	0.989	0.989
44	0.989	0.989	0.989	0.989	0.989	0.989
45	0.977	0.976	0.977	0.976	0.977	0.976
46	0.982	0.980	0.982	0.980	0.982	0.980
47	0.985	0.983	0.985	0.983	0.985	0.983
48	0.987	0.985	0.987	0.985	0.987	0.985
49	0.989	0.987	0.989	0.987	0.989	0.987
50	0.990	0.988	0.990	0.988	0.990	0.988
51	0.991	0.989	0.991	0.989	0.991	0.989
52	0.992	0.990	0.992	0.990	0.992	0.990
53	0.993	0.991	0.993	0.991	0.993	0.991
54	0.993	0.992	0.993	0.992	0.993	0.992
55	0.983	0.981	0.983	0.981	0.983	0.981
56	0.984	0.982	0.984	0.982	0.984	0.982
57	0.985	0.984	0.985	0.984	0.985	0.984
58	0.986	0.984	0.986	0.984	0.986	0.984
59	0.987	0.985	0.987	0.985	0.987	0.985
60	0.988	0.986	0.988	0.986	0.988	0.986
61	0.988	0.987	0.988	0.987	0.988	0.987
62	0.989	0.987	0.989	0.987	0.989	0.987
63	0.989	0.988	0.989	0.988	0.989	0.988
64	0.990	0.988	0.990	0.988	0.990	0.988
65	0.977	0.970	0.977	0.970	0.977	0.970

66	0.980	0.974	0.980	0.974	0.980	0.974
67	0.982	0.977	0.982	0.977	0.982	0.977
68	0.983	0.979	0.983	0.979	0.983	0.979
69	0.985	0.981	0.985	0.981	0.985	0.981
70	0.986	0.983	0.986	0.983	0.986	0.983
71	0.987	0.984	0.987	0.984	0.987	0.984
72	0.988	0.985	0.988	0.985	0.988	0.985
73	0.989	0.986	0.989	0.986	0.989	0.986
74	0.989	0.987	0.989	0.987	0.989	0.987
75	0.960	0.949	0.960	0.949	0.960	0.949
76	0.964	0.956	0.964	0.956	0.964	0.956
77	0.967	0.962	0.967	0.962	0.967	0.962
78	0.969	0.966	0.969	0.966	0.969	0.966
79	0.972	0.969	0.972	0.969	0.972	0.969
80	0.973	0.972	0.973	0.972	0.973	0.972
81	0.975	0.974	0.975	0.974	0.975	0.974
82	0.977	0.976	0.977	0.976	0.977	0.976
83	0.978	0.978	0.978	0.978	0.978	0.978
84	0.979	0.979	0.979	0.979	0.979	0.979
85	0.935	0.922	0.935	0.922	0.935	0.922
86	0.938	0.926	0.938	0.926	0.938	0.926
87	0.941	0.930	0.941	0.930	0.941	0.930
88	0.944	0.933	0.944	0.933	0.944	0.933
89	0.946	0.936	0.946	0.936	0.946	0.936
90	0.948	0.939	0.948	0.939	0.948	0.939
91	0.950	0.942	0.950	0.942	0.950	0.942
92	0.951	0.944	0.951	0.944	0.951	0.944
93	0.953	0.946	0.953	0.946	0.953	0.946
94	0.955	0.948	0.955	0.948	0.955	0.948
95	0.956	0.950	0.956	0.950	0.956	0.950
96	0.957	0.951	0.957	0.951	0.957	0.951
97	0.958	0.953	0.958	0.953	0.958	0.953
98	0.960	0.954	0.960	0.954	0.960	0.954
99	0.961	0.956	0.961	0.956	0.961	0.956
100	0.962	0.957	0.962	0.957	0.962	0.957
101	0.963	0.958	0.963	0.958	0.963	0.958

102	0.964	0.959	0.964	0.959	0.964	0.959
103	0.964	0.960	0.964	0.960	0.964	0.960
104	0.965	0.961	0.965	0.961	0.965	0.961
105	0.966	0.962	0.966	0.962	0.966	0.962
106	0.967	0.963	0.967	0.963	0.967	0.963
107	0.967	0.964	0.967	0.964	0.967	0.964
108	0.968	0.965	0.968	0.965	0.968	0.965
109	0.969	0.965	0.969	0.965	0.969	0.965
109+	0.969	0.965	0.969	0.965	0.969	0.965

Breast Cancer

Table 18. Probability of 1, 5 year survival data for Breast cancer(16)

Age	Survival probability – 1 year		Survival probability – 5 year	
	M	F	M	F
50-59	N/A	0.987	N/A	0.923
60-69	N/A	0.984	N/A	0.927
70-79	N/A	0.973	N/A	0.909
80-99	N/A	0.898	N/A	0.741

Colorectal Cancer

Table 19. Probability of 1, 5 year survival data for Colorectal cancer(16)

Age	Survival probability – 1 year		Survival probability – 5 year	
	M	F	M	F
15-44	0.876	0.887	0.707	0.713
45-54	0.86	0.867	0.634	0.665
55-64	0.858	0.860	0.672	0.679
65-74	0.827	0.813	0.661	0.651
75+	0.679	0.623	0.49	0.464

Endometrial Cancer

Table 20. Probability of 1, 5 year survival for Endometrial cancer(16)

Age	Survival probability – 1 year		Survival probability – 5 year	
	M	F	M	F
15-44		0.946		0.876
45-54	NA	0.945	NA	0.869
55-64	NA	0.948	NA	0.855
65-74	NA	0.915	NA	0.785
75+	NA	0.807	NA	0.631

Kidney Cancer

Table 21. Probability of 1, 5 year survival data for Kidney cancer(16)

Age	Survival probability – 1 year		Survival probability – 5 year	
	M	F	M	F
15-49	0.898	0.913	0.804	0.84
50-59	0.855	0.873	0.721	0.781
60-69	0.817	0.842	0.661	0.717
70-79	0.785	0.797	0.611	0.654
80-99	0.651	0.619	0.476	0.442

Oesophagus Cancer

Table 22. Probability of 1, 5 year survival data for Oesophageal cancer(16)

Age	Survival probability – 1 year		Survival probability – 5 year	
	M	F	M	F
15-49	0.559	0.531	0.227	NA
50-59	0.537	0.589	0.227	0.248
60-69	0.524	0.543	0.204	0.257
70-79	0.501	0.519	0.197	0.211
80-99	0.345	0.293	0.094	0.083

Ovarian Cancer

Table 23. Probability of 1, 5 year survival data for Ovarian cancer(16)

Age	Survival probability – 1 year		Survival probability – 5 year	
	M	F	M	F
15-49	NA	0.946	NA	0.847
50-59	NA	0.920	NA	0.692
60-69	NA	0.847	NA	0.556
70-79	NA	0.757	NA	0.423
80-99	NA	0.484	NA	0.246

Pancreas Cancer

Table 24. Probability of 1, 5 year data for Oesophagus cancer(16)

Age	Survival probability – 1 year		Survival probability – 5 year	
	M	F	M	F
15-49	0.477	0.655	0.917	NA
50-59	0.356	0.400	0.125	0.178
60-69	0.292	0.320	0.076	0.093
70-79	0.231	0.259	0.056	0.060
80-99	0.131	0.124	0.033	0.034

Relative Risks

This document provides the sources and estimates of the Relative Risk (RR) of defined diseases according to BMI status (given as per BMI unit increase from BMI 22 = 1.0; and per BMI category of Overweight and Obese relative to normal weight). The RR used for the Dynamo-HIA study is given in the last line of each table. Adjustments for age and smoking are given as multipliers of the differential risk, i.e. as a multiplier of the difference in relative risk from the base (1.0). Thus an adjustment multiplier of x0.95 applied to an RR of 1.20 would lead to an RR of 1.19 (calculated as RR' = 1 + A(RR-1) where RR is the given relative risk, RR' is the adjusted relative risk and A is the adjustment multiplier).

Table 25: Relative risk for Breast cancer

BMI groups (kg/m^2)	Breast cancer Age groups 0-49	0-49	50-110	50-110
	M	F	M	F
<25	1.000	1.000	1.000	1.000
25-30	1.000	1.000	1.000	1.120
>30	1.000	1.000	1.000	1.250

Table 26: Relative risk for Coronary heart disease

BMI groups (kg/m^2)	Coronary heart disease Age groups 20-64	20-64	65-110	65-110
	M	F	M	F
22	1.000	1.000	1.000	1.000
23	1.070	1.100	1.049	1.070
24	1.145	1.210	1.101	1.147
25	1.225	1.331	1.158	1.232
26	1.311	1.464	1.218	1.325
27	1.403	1.611	1.282	1.427
28	1.501	1.772	1.351	1.540
29	1.606	1.949	1.424	1.664
30	1.718	2.144	1.503	1.801
31	1.838	2.358	1.587	1.951
32	1.967	2.594	1.677	2.116
33	2.105	2.853	1.773	2.297
34	2.252	3.138	1.877	2.497
35	2.410	3.452	1.987	2.717
36	2.579	3.797	2.105	2.958
37	2.759	4.177	2.231	3.224
38	2.952	4.595	2.367	3.516
39	3.159	5.054	2.511	3.838
40	3.380	5.560	2.666	4.192
41	3.617	6.116	2.832	4.581
42	3.870	6.727	3.009	5.009
43	4.141	7.400	3.198	5.480

44	4.430	8.140	3.401	5.998
45+	4.741	8.954	3.618	6.568

Table 27: Relative risks for Colorectal cancer

BMI groups (kg/m^2)	Colorectal cancer			
	Age groups			
	20-44	20-44	45-110	45-110
	M	F	M	F
22	1.000	1.000	1.000	1.000
23	1.040	1.020	1.036	1.018
24	1.082	1.040	1.073	1.036
25	1.125	1.061	1.112	1.055
26	1.170	1.082	1.153	1.074
27	1.217	1.104	1.195	1.094
28	1.265	1.126	1.239	1.114
29	1.316	1.149	1.284	1.134
30	1.369	1.172	1.332	1.154
31	1.423	1.195	1.381	1.176
32	1.480	1.219	1.432	1.197
33	1.539	1.243	1.486	1.219
34	1.601	1.268	1.541	1.241
35	1.665	1.294	1.599	1.264
36	1.732	1.319	1.659	1.288
37	1.801	1.346	1.721	1.311
38	1.873	1.373	1.786	1.336
39	1.948	1.400	1.853	1.360
40	2.026	1.428	1.923	1.385
41	2.107	1.457	1.996	1.411
42	2.191	1.486	2.072	1.437
43	2.279	1.516	2.151	1.464
44	2.370	1.546	2.233	1.491
45+	2.465	1.577	2.318	1.519

Table 28: Relative risk for Diabetes (Type 2)

	Diabetes (from no disease to diabetes)
	Age groups

BMI groups (kg/m²)	20-64	20-64	65-74	65-74	75-110	75-110
	M	F	M	F	M	F
22	1.000	1.000	1.000	1.000	1.000	1.000
23	1.180	1.220	1.166	1.202	1.149	1.182
24	1.392	1.488	1.361	1.449	1.325	1.404
25	1.643	1.816	1.592	1.751	1.532	1.676
26	1.939	2.215	1.864	2.118	1.777	2.006
27	2.288	2.703	2.185	2.566	2.066	2.410
28	2.700	3.297	2.564	3.114	2.407	2.902
29	3.185	4.023	3.011	3.781	2.810	3.503
30	3.759	4.908	3.538	4.595	3.284	4.236
31	4.435	5.987	4.161	5.588	3.845	5.130
32	5.234	7.305	4.895	6.800	4.506	6.220
33	6.176	8.912	5.762	8.279	5.286	7.551
34	7.288	10.872	6.785	10.082	6.206	9.174
35	8.599	13.264	7.991	12.283	7.292	11.155
36	10.147	16.182	9.415	14.968	8.574	13.571
37	11.974	19.742	11.096	18.243	10.086	16.519
38	14.129	24.086	13.079	22.239	11.871	20.115
39	16.672	29.384	15.418	27.114	13.977	24.502
40	19.673	35.849	18.179	33.061	16.461	29.855
41	23.214	43.736	21.437	40.317	19.394	36.385
42	27.393	53.358	25.282	49.169	22.853	44.352
43	32.324	65.096	29.818	59.969	26.936	54.072
44	38.142	79.418	35.171	73.144	31.754	65.930
45+	45.008	96.889	41.487	89.218	37.438	80.396

Table 29: Relative risks for Endometrial cancer

BMI groups (kg/m²)	Endometrial cancer			
	Age groups			
	0-19	0-19	20-110	20-110
	M	F	M	F
22	1.000	1.000	1.000	1.000
23	1.000	1.000	1.000	1.100
24	1.000	1.000	1.000	1.210

25	1.000	1.000	1.000	1.331
26	1.000	1.000	1.000	1.464
27	1.000	1.000	1.000	1.611
28	1.000	1.000	1.000	1.772
29	1.000	1.000	1.000	1.949
30	1.000	1.000	1.000	2.144
31	1.000	1.000	1.000	2.358
32	1.000	1.000	1.000	2.594
33	1.000	1.000	1.000	2.853
34	1.000	1.000	1.000	3.138
35	1.000	1.000	1.000	3.452
36	1.000	1.000	1.000	3.797
37	1.000	1.000	1.000	4.177
38	1.000	1.000	1.000	4.595
39	1.000	1.000	1.000	5.054
40	1.000	1.000	1.000	5.560
41	1.000	1.000	1.000	6.116
42	1.000	1.000	1.000	6.727
43	1.000	1.000	1.000	7.400
44	1.000	1.000	1.000	8.140
45+	1.000	1.000	1.000	8.954

Table 30:Relative risks for Hypertension

BMI groups (kg/m^2)	Hypertension			
	Age groups			
	0-20	0-20	20-110	20-110
	M	F	M	F
15-24.9	1.000	1.000	1.000	1.000
25-29	1.000	1.000	1.880	1.880
30-39.9	1.000	1.000	3.720	3.720
>40	1.880	1.000	7.030	7.030

Table 31: Relative risks for Knee Osteoarthritis

BMI groups (kg/m^2)	Knee Osteoarthritis			
	Age groups			
	16-75	16-75	76-110	76-110

	M	F	M	F
<25	1.000	1.000	1.000	1.000
25-30	2.450	2.450	1.000	1.000
>30	4.550	4.550	1.000	1.000

Table 32: Relative risks for Oesophageal cancer

BMI groups (kg/m^2)	Oesophageal cancer			
	Age Groups			
	0-24	0-24	25-100	25-100
	M	F	M	F
22	1.000	1.000	1.000	1.000
23	1.000	1.000	1.100	1.080
24	1.000	1.000	1.210	1.166
25	1.000	1.000	1.331	1.260
26	1.000	1.000	1.464	1.360
27	1.000	1.000	1.611	1.469
28	1.000	1.000	1.772	1.587
29	1.000	1.000	1.949	1.714
30	1.000	1.000	2.144	1.851
31	1.000	1.000	2.358	1.999
32	1.000	1.000	2.594	2.159
33	1.000	1.000	2.853	2.332
34	1.000	1.000	3.138	2.518
35	1.000	1.000	3.452	2.720
36	1.000	1.000	3.797	2.937
37	1.000	1.000	4.177	3.172
38	1.000	1.000	4.595	3.426
39	1.000	1.000	5.054	3.700
40	1.000	1.000	5.560	3.996
41	1.000	1.000	6.116	4.316
42	1.000	1.000	6.727	4.661
43	1.000	1.000	7.400	5.034
44	1.000	1.000	8.140	5.437
45+	1.000	1.000	8.954	5.871

Table 33: Relative risks for Ovarian cancer

BMI groups (kg/m²)	Ovarian cancer			
	Age groups			
	0-17	0-17	18-100	18-100
	M	F	M	F
<22.5	1.000	1.000	1.000	1.000
22.5-25	1.000	1.000	1.000	1.010
25-27.5	1.000	1.000	1.000	1.050
27.5-30	1.000	1.000	1.000	1.100
30-32.5	1.000	1.000	1.000	1.170
<32.5	1.000	1.000	1.000	1.280

Table 34: Relative risks for Pancreatic cancer

BMI groups (kg/m²)	Pancreatic cancer			
	Age groups			
	0-17	0-17	18-100	18-100
	M	F	M	F
<25	1.000	1.000	1.000	1.000
25-30	1.000	1.000	1.140	1.140
>30	1.000	1.000	1.300	1.300

Table 35: Relative risks for Renal cancer

BMI groups (kg/m²)	Renal cancer			
	Age groups			
	0-19	0-19	20-110	20-110
	M	F	M	F
22	1.000	1.000	1.000	1.000
23	1.000	1.000	1.050	1.050
24	1.000	1.000	1.103	1.103
25	1.000	1.000	1.158	1.158
26	1.000	1.000	1.216	1.216
27	1.000	1.000	1.276	1.276
28	1.000	1.000	1.340	1.340
29	1.000	1.000	1.407	1.407
30	1.000	1.000	1.477	1.477
31	1.000	1.000	1.551	1.551
32	1.000	1.000	1.629	1.629

33	1.000	1.000	1.710	1.710
34	1.000	1.000	1.796	1.796
35	1.000	1.000	1.886	1.886
36	1.000	1.000	1.980	1.980
37	1.000	1.000	2.079	2.079
38	1.000	1.000	2.183	2.183
39	1.000	1.000	2.292	2.292
40	1.000	1.000	2.407	2.407
41	1.000	1.000	2.527	2.527
42	1.000	1.000	2.653	2.653
43	1.000	1.000	2.786	2.786
44	1.000	1.000	2.925	2.925
45+	1.000	1.000	3.072	3.072

Table 36: Relative risks for Stroke

BMI groups (kg/m^2)	Stroke			
	Age groups			
	20-64	20-64	65-110	65-110
	M	F	M	F
22	1.000	1.000	1.000	1.000
23	1.040	1.040	1.030	1.030
24	1.082	1.082	1.061	1.061
25	1.125	1.125	1.094	1.094
26	1.170	1.170	1.127	1.127
27	1.217	1.217	1.162	1.162
28	1.265	1.265	1.199	1.199
29	1.316	1.316	1.237	1.237
30	1.369	1.369	1.276	1.276
31	1.423	1.423	1.317	1.317
32	1.480	1.480	1.360	1.360
33	1.539	1.539	1.405	1.405
34	1.601	1.601	1.451	1.451
35	1.665	1.665	1.499	1.499
36	1.732	1.732	1.549	1.549
37	1.801	1.801	1.601	1.601
38	1.873	1.873	1.655	1.655

39	1.948	1.948	1.711	1.711
40	2.026	2.026	1.769	1.769
41	2.107	2.107	1.830	1.830
42	2.191	2.191	1.893	1.893
43	2.279	2.279	1.959	1.959
44	2.370	2.370	2.027	2.027
45+	2.465	2.465	2.099	2.099

Health economic data

Sources of cost data

UKHF conducted a review of current literature to identify the direct costs associated with treatments and services for specific health conditions that are covered by public funds. NHS 2012/2013 programme budget costs were used when no data was public data available. The main types of costs that were included are defined briefly below, however please note that the not all studies had all of these costs included in their research:

- Primary care is often the primary point of contact of someone seeking care. GP visits are the main source, but studies, when available, include other types of services offered by most of the GP practices. These include nurse visits, home visits, phone/email/fax consultations.
- Prescription costs are usually estimated as the volume times the costs of primary care prescription.
- Inpatient costs are the total costs of treating a patient at hospital for a specific diagnosis (episode). They include day cases, elective and emergency admissions.
- Outpatient costs capture the costs of visits to specialists.

We have not included in our different costs indirect costs such as the loss of income when hospitalised.

Summary of identified costs

Table 1 summarises the costs used in the microsimulation. The majority of costs were extracted from the literature, however costs for two diseases came from the 2012-13 NHS programme budget costs.[1] More information about the costs used in each paper can be found in Health Economic Review excel workbook.

[1]We have been advised that Programme Budgeting data does not fully capture the actual health care expenditures, in particular for social care costs. Thus costs from the literature were preferred where data exists.

All the costs were adjusted using prevalence when necessary to represent the total cost per type of care and per disease group for England.

For the microsimulation model, we need the cost per case, which is the total cost divided by the prevalence in 2016. Therefore this figure is not necessarily equal to the unit cost as patients use different combinations and quantities of care.

Relevant sources were collated using a systematic literature review in PubMed, and completed using Google searches. We searched for peer-reviewed articles using PubMed. We also used Google to identify reports from other sources. We focused exclusively on costs based on English or UK data. While multiple studies from the search results were considered, the most relevant, recent studies were used for the final cost estimates. We rarely had the choice between two references, but in this case our selection criteria were the transparency of the method to estimate the costs with a preference for bottom-up approaches,[2] the clarity of the methodology and definitions, the source of data with a preference for national representative samples, and the years for which the costs were reported. All costs were adjusted for inflation using the CCEMG-EPPI-Centre cost Converter and divided by the prevalence in order to have a “cost per case”.(1)

[2] A bottom-up approach, in contrast to a top-down approach, reflects the actual needs. It quantifies each resource required to provide the services or treatments to care for patients with a specific condition, multiplied by the input costs. A top-down approach allocates a total figure (e.g. the NHS programme budgeting cost) to different services as such is less likely to capture the actual spending associated with a specific disease.

Disease	Cost per case (£) (inflated to 2016 values)	Outpatient (post diagnosis)	Hospital	Prescription	Year	Method	Reference	Location
Colorectal cancer	£13563.22	□	□	□	2011-2012	Bottom-up	Hall et al 2015	UK
Oesophageal cancer	£9568.28	□	□	□	2006-2007	Bottom-up	Agus et al. 2013	NI
Renal (kidney) cancer	£414.81	□	□	□	2012-2013		NHS programme budget	England
Ovarian cancer	£1408.94				2012-2013		NHS programme budget	England
Pancreatic cancer	£5735.93	□	□	□	2008	Bottom-up	Mauro Laudicello and Imperial College with Pancreatic Cancer UK.	UK
CHD	£2838.70	□	□	□	1999	Top down	Various	England
Stroke	£1627.26	□	□	□	2005	Bottom-up	Saka et al. 2009	England
Type 2 Diabetes	£672.28	□	□	□	2010-2011	Top down	Various	England

Hypertension	£493.15	□	✓ - tests not stay	□	2007-2008	Bottom-up	Brilleman et al 2013	UK
Knee osteoarthritis	£223.97	□	□	□	2010	Bottom-up	Chen et al 2012	UK
Endometrial cancer	£2471.21	□	□	□	2012-2013	Bottom-up	Pennington et al 2016	England
Breast cancer	£13295.53	□	□	□	2011-2012	Bottom-up	Hall et al. (2015).	UK

Limitations

The main overall limitation of using costs from the literature is that the estimation methods vary significantly from one paper to another, and the inputs for each category of care are slightly different for each condition. When the estimates come from a bottom-up approach, the costs are likely to underestimate the true costs as the possible missing components are set to zero. When the estimates come from a top-down approach, the allocation rule is often not clear and it is hard to know how comparable they are to the true cost. Yet, the order of magnitude is likely to represent the true costs for the NHS, as the overall costs are broken down into parts. Furthermore, the authors often argue that their method is conservative and that the estimated costs represent lower-bound estimates.

Utility weights

All utility weightings for use in QALY calculations were obtained from Sullivan et al's 2011 Catalogue of EQ-5D scores for the United Kingdom and NICE (20)

Males and females were allocated the same EQ-5D score, as this is not specified by gender in the publication. The diseases were mapped onto conditions listed in the publication using matching, or closest matching ICD9 and Clinical Classification Categories.

Table 5 - List of EQ-5D values allocated to males and females for each disease

Disease	Male	Female	Source
Breast cancer	N/A	0.749	Sullivan et al. 2011(21)
CHD	0.76	0.76	Laires et al. 2015 (22)
Colorectal cancer	0.676	0.676	Sullivan et al. 2011(21)
Diabetes (Type 2)	0.661	0.661	Sullivan et al. 2011(21)
Endometrial cancer	N/A	0.598	Sullivan et al. 2011(21)
Hypertension	0.721	0.721	Sullivan et al. 2011(21)
Knee Osteoarthritis	0.49	0.46	Conner-Spady et al. 2015 (23)
Oesophageal cancer	0.904	0.904	Sullivan et al. 2011(21)
Ovarian cancer	N/A	0.848	Sullivan et al. 2011(21)
Pancreatic cancer	0.79	0.79	Romanus et al. 2012 (24)
Renal cancer	0.661	0.661	Sullivan et al. 2011(21)
Stroke	0.713	0.713	Rivero-Arias et al. 2010 (25)

UKHF microsimulation methodology

Microsimulation framework

Our simulation consists of two modules. The first module calculates the predictions of risk factor trends over time based on data from rolling cross-sectional studies. The second module performs the microsimulation of a virtual population, generated with demographic characteristics matching those of the observed data. The health trajectory of each individual from the population is simulated over time allowing them to contract, survive or die from a set of diseases or injuries related to the analysed risk factors. The detailed description of the two modules is presented below.

Microsimulation Module one: Predictions of overweight/obesity over time

BMI was analysed within the model as risk factors (RF), as described in Table 6.

Table 6 Description of the categories used for BMI risk factors

Risk factor (RF)	Number of categories (N)	Categories
BMI	5	1 Normal weight BMI < 25 kg m^{-2} (normal weight)<br>2 Overweight BMI from 25 to 29.99 kg m^{-2} (overweight)<br>3 Obese BMI ≥ 30 kg m^{-2} (obesity class I & class II & class III)

Let K be the number of categories for BMI, e.g. $K=3$ in this paper. Let $k=1,2,\ldots,K$ number these categories and $p_k(t)$ denote the prevalence of individuals with BMI values that correspond to the category k at time t .

We estimate $p_k(t)$ using multinomial logistic model with time t as a single explanatory variable. In the first step, for $k > 1$, we have

$$\ln\left(\frac{p_k(t)}{p_1(t)}\right)=a_k+b_kt \qquad (0.1)$$

The prevalence of the third category, $p_k(t)$, is obtained by using the normalisation constraint $\sum_{k=1}^{K} p_k(t)=1$ Solving equation (0.1) for $p_k(t)$, we obtain

$$p_k(t) = \frac{\exp(a_k + b_k t)}{1 + \sum_{k=2}^{K} \exp(a_k + b_k t)}, \qquad (0.2)$$

which is subjected to all constraints on the prevalence values, i.e. normalisation and [0, 1] bounds.

Multinomial logistic regression for each risk factor

Measured data is extracted from the survey data set. They consist of sets of probabilities with their variances. Each set represents the probabilities of individuals of normal weight, overweight, obesity class I & class II and obesity class III at specific time values (i.e., the year of the survey). For any particular time the sum of these probabilities is unity.

Each data point is treated as a normally distributed random variable; together they are a set of N groups (number of years) of K probabilities $\{\{t_i, \mu_{ki}, \sigma_{ki} \mid k \in [1, K]\} \mid i \in [1, N]\}$, where t_i, μ_{ki}, σ_{ki} denote the year of the survey, the mean probability of $k-th$ BMI category of the year and its variance respectively.

The regression consists of fitting a set of logistic functions $\{p_k(a,b,t) \mid k \in [1, K]\}$ to these data – one function for each k-value. At each time value the sum of these functions is unity. Thus, for example, when measuring obesity in the four states, the $k = 1$ regression function represents the probability of being normal weight over time, $k = 2$ the probability of being overweight, $k = 3$ the probability of being of obesity class I & class II & class III.

$$S(\mathbf{a},\mathbf{b}) = \tfrac{1}{2} \sum_{k=0}^{k=K-1} \sum_{i=0}^{i=N-1} \frac{\left(p_k(\mathbf{a},\mathbf{b};t_i) - \mu_{ki}\right)^2}{\sigma_{ki}^2} \qquad (0.2)$$

$$\begin{gathered} p_k(\mathbf{a},\mathbf{b},t) \equiv \frac{e^{A_k}}{1 + e^{A_1} + .. + e^{A_{K-1}}} \\ \mathbf{a} \equiv (a_0, a_1, .., a_{K-1}), \quad \mathbf{b} \equiv (b_0, b_1, .., b_{K\text{-}1}) \\ A_0 \equiv 0, \quad A_k \equiv a_k + b_k t \end{gathered} \qquad (0.2)$$

The parameters A_0, a_0 and b_0 are all zero and are used merely to preserve the symmetry of the expressions and their manipulation. For a K-dimensional set of probabilities there will be $2(K-1)$ regression parameters to be determined due to the normalisation constraint.

The minimum of the function S is determined from the equations

$$\frac{\partial S}{\partial a_j} = \frac{\partial S}{\partial b_j} = 0 \qquad \text{for j=1,2,....,k-1} \tag{0.2}$$

noting the relations

$$\frac{\partial p_k}{\partial A_j} = \frac{\partial}{\partial A_j}\left(\frac{e^{A_k}}{1+e^{A_1}+..+e^{A_{K-1}}}\right) = p_k\delta_{kj} - p_k p_j$$
$$\frac{\partial}{\partial a_j} = \frac{\partial}{\partial A_j} \tag{0.3}$$
$$\frac{\partial}{\partial b_j} = t\frac{\partial}{\partial A_j}$$

The values of the vectors a, b that satisfy these equations are denoted $\hat{\mathbf{a}}$, $\hat{\mathbf{b}}$ respectively. They provide the trend lines, $p_k\left(\hat{\mathbf{a}},\hat{\mathbf{b}};t\right)$, for the probabilities of each BMI category. The confidence intervals for the trend lines are derived most easily from the underlying Bayesian analysis of the problem.

Bayesian interpretation

The $(2K-2)$ regression parameters $\{\mathbf{a},\mathbf{b}\}$ are regarded as random variables whose posterior distribution is proportional to the function $exp\left(-S\left(\mathbf{a},\mathbf{b}\right)\right)$. The maximum likelihood estimate of this probability distribution function, the minimum of the function S, is obtained at the values $\hat{\mathbf{a}}$, $\hat{\mathbf{b}}$. Other properties of the $(2K-2)-$dimensional probability distribution function are obtained by first approximating it as a $(2K-2)-$dimensional normal distribution whose mean is the maximum likelihood estimate. This amounts to expanding the function $S(\mathbf{a},\mathbf{b})$ in a Taylor series as far as terms quadratic in the differences $(\mathbf{a}-\hat{\mathbf{a}})$, $(\mathbf{b}-\hat{\mathbf{b}})$ about the maximum likelihood estimate $\hat{\mathbf{S}} \equiv S\left(\hat{\mathbf{a}},\hat{\mathbf{b}}\right)$.

Hence

$$
\begin{aligned}
S(\mathbf{a},\mathbf{b}) &= \tfrac{1}{2}\sum_{k=0}^{k=K-1}\sum_{i=0}^{i=N-1}\frac{\left(p_k(\mathbf{a},\mathbf{b};t_i)-\mu_{ki}\right)^2}{\sigma_{ki}^2} \\
&\equiv S\left(\hat{\mathbf{a}},\hat{\mathbf{b}}\right)+\tfrac{1}{2}\left(a-\hat{a},b-\hat{b}\right)P^{-1}\left(a-\hat{a},b-\hat{b}\right)+\ldots \\
&\approx S\left(\hat{\mathbf{a}},\hat{\mathbf{b}}\right)+\tfrac{1}{2}\sum_{i,j}(a_i-\hat{a}_i)\frac{\partial^2\hat{S}}{\partial\hat{a}_i\partial\hat{a}_j}\left(a_j-\hat{a}_j\right)+\tfrac{1}{2}\sum_{i,j}(a_i-\hat{a}_i)\frac{\partial^2\hat{S}}{\partial\hat{a}_i\partial\hat{b}_j}\left(b_j-\hat{b}_j\right)+ \\
&+\tfrac{1}{2}\sum_{i,j}\left(b_i-\hat{b}_i\right)\frac{\partial^2\hat{S}}{\partial\hat{b}_i\partial\hat{a}_j}\left(a_j-\hat{a}_j\right)+\tfrac{1}{2}\sum_{i,j}\left(b_i-\hat{b}_i\right)\frac{\partial^2\hat{S}}{\partial\hat{b}_i\partial\hat{b}_j}\left(b_j-\hat{b}_j\right)
\end{aligned}
\qquad (0.3)
$$

The $(2K-2)-$dimensional covariance matrix P is the inverse of the appropriate expansion coefficients. This matrix is central to the construction of the confidence limits for the trend lines.

Estimation of the confidence intervals

The logistic regression functions $p_k(t)$ can be approximated as a normally distributed time-varying random variable $N\left(\hat{p}_k(t),\sigma_k^2(t)\right)$ by expanding p_k about its maximum likelihood estimate (the trend line) $\hat{p}_k(t)=p\left(\hat{\mathbf{a}},\hat{\mathbf{b}},t\right)$

$$
\begin{aligned}
p_k(\mathbf{a},\mathbf{b},t) &= p_k\left(\hat{\mathbf{a}}+\mathbf{a}-\hat{\mathbf{a}},\hat{\mathbf{b}}+\mathbf{b}-\hat{\mathbf{b}},t\right) \\
&= \hat{p}_k(t)+\left(\nabla_{\hat{a}},\nabla_{\hat{b}}\right)\hat{p}_k(t)\begin{pmatrix}\mathbf{a}-\hat{\mathbf{a}}\\ \mathbf{b}-\hat{\mathbf{b}}\end{pmatrix}+\ldots
\end{aligned}
\qquad (0.3)
$$

Denoting mean values by angled brackets, the variance of p_k is thereby approximated as

$$
\begin{aligned}
&\sigma_k^2(t)\equiv\left\langle\left(p_k(\mathbf{a},\mathbf{b},t)-\hat{p}_k(t)\right)^2\right\rangle=\left(\nabla_{\hat{a}}\hat{p}_k(t),\nabla_{\hat{b}}\hat{p}_k(t)\right)\left\langle\begin{pmatrix}\mathbf{a}-\hat{\mathbf{a}}\\ \mathbf{b}-\hat{\mathbf{b}}\end{pmatrix}\begin{pmatrix}\mathbf{a}-\hat{\mathbf{a}}\\ \mathbf{b}-\hat{\mathbf{b}}\end{pmatrix}^T\right\rangle\times \\
&\left(\nabla_{\hat{a}}\hat{p}_k(t),\nabla_{\hat{b}}\hat{p}_k(t)\right)^T=\left(\nabla_{\hat{a}}\hat{p}_k(t),\nabla_{\hat{b}}\hat{p}_k(t)\right)\ P\ \left(\nabla_{\hat{a}}\hat{p}_k(t),\nabla_{\hat{b}}\hat{p}_k(t)\right)^T
\end{aligned}
\qquad (0.3)
$$

When $K=3$ this equation can be written as the 4-dimensional inner product

$$
\sigma_k^2(t)=\begin{pmatrix}\dfrac{\partial\hat{p}_k(t)}{\partial\hat{a}_1} & \dfrac{\partial\hat{p}_k(t)}{\partial\hat{a}_2} & \dfrac{\partial\hat{p}_k(t)}{\partial\hat{b}_1} & \dfrac{\partial\hat{p}_k(t)}{\partial\hat{b}_2}\end{pmatrix}\begin{bmatrix}P_{aa11} & P_{aa12} & P_{ab11} & P_{ab12}\\ P_{aa21} & P_{aa22} & P_{ab21} & P_{ab22}\\ P_{ba11} & P_{ba12} & P_{bb11} & P_{bb12}\\ P_{ba21} & P_{ba22} & P_{bb21} & P_{bb22}\end{bmatrix}\begin{pmatrix}\dfrac{\partial\hat{p}_k(t)}{\partial\hat{a}_1}\\ \dfrac{\partial\hat{p}_k(t)}{\partial\hat{a}_2}\\ \dfrac{\partial\hat{p}_k(t)}{\partial\hat{b}_1}\\ \dfrac{\partial\hat{p}_k(t)}{\partial\hat{b}_2}\end{pmatrix}
\qquad (0.3)
$$

where $P_{cdij} \equiv \left\langle (c_i - \hat{c}_i)\left(d_j - \hat{d}_j\right)\right\rangle$. The 95% confidence interval for $p_k(t)$ is centred given as $\left[\hat{p}_k(t) - 1.96\sigma_k(t),\ \mathrm{p}_\mathrm{k}(t) + 1.96\sigma_k(t)\right]$.

Module two: Microsimulation model

Microsimulation initialisation: birth, disease and death models

Simulated people are generated with the correct demographic statistics in the simulation's start-year. In this year women are stochastically allocated the number and years of birth of their children – these are generated from known fertility and mother's age at birth statistics (valid in the start-year). If a woman has children then those children are generated as members of the simulation in the appropriate birth year.

The microsimulation is provided with a list of BMI-related diseases. These diseases used the best available incidence, mortality, survival, relative risk and prevalence statistics (by age and gender). Individuals in the model are simulated from the start year of the simulation. In the course of their lives, simulated people can die from one of the diseases caused by BMI that they might have acquired or from some other cause(s). The probability that a person of a given age and gender dies from a cause other than the disease are calculated in terms of known death and disease statistics valid in the start-year. It is constant over the course of the simulation.

The microsimulation incorporates a sophisticated economic module. The module employs a Markov-type simulation of long-term health benefits and health care costs. It synthesises and estimates evidence on cost-utility analysis. The model is used to project the differences in quality-adjusted life years (QALYs), direct lifetime health-care costs, adult productivity costs, Lifetime Income Losses costs, total diability-adjusted life years (DALY) and incremental cost-effectiveness ratio over a specified time scale. The direct healthcare costs are presented separately in terms of hospital admissions, general practitioner costs, medication costs and social care costs. Adult productivity costs are presented as absenteeism costs incurred each year or income losses due to premature mortality each year. Lifetime Income Losses costs each year is the proportion of lifetime income lost due to individuals being overweight or obese in childhood. Outputs can be discounted for any specific discount rate.

This following section provides an overview of the main assumptions of the model.

Population models

Populations are implemented as instances of the TPopulation C++ class. The TPopulation class is created from a population (*.ppl) file. Usually a simulation will use only one population but it can

simultaneously process multiple populations (for example, different ethnicities within a national population).

Population Editor

The Population Editor Allows editing and testing of TPopulation objects. The population is created in the start-year and propagated forwards in time. An example population pyramid which can be used when initialising the model is shown in Figure 1 shows the population distribution for England in 2016 used in the initialisation of the model.

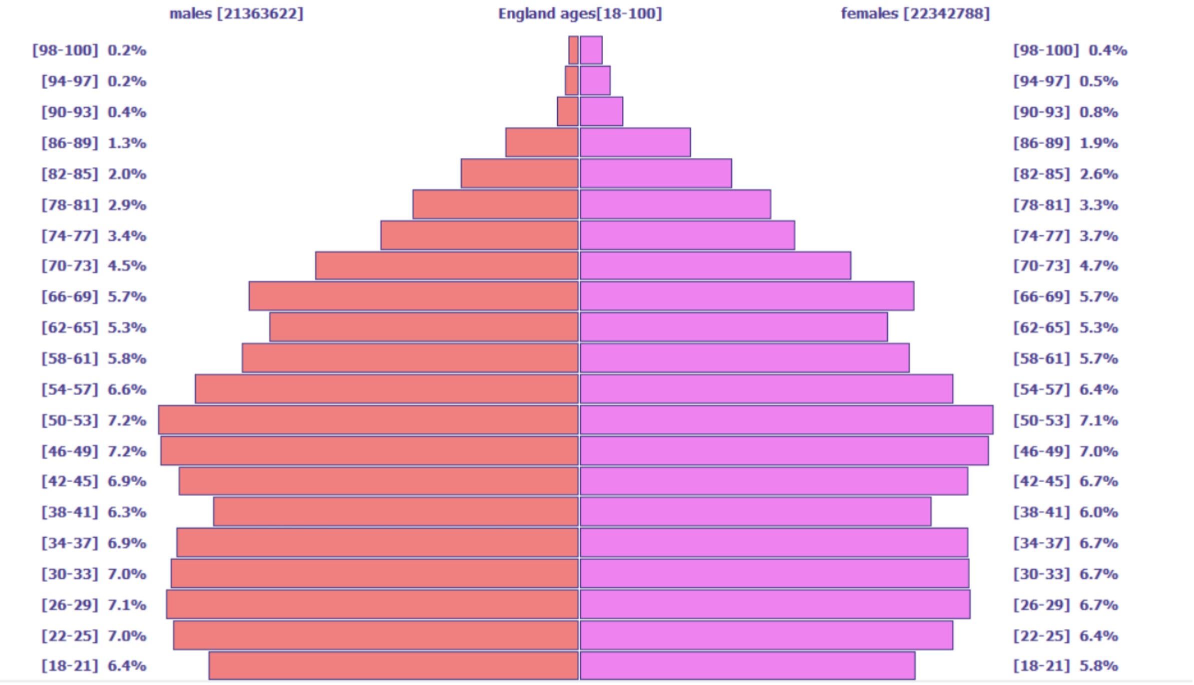

Figure 1 Population pyramid for England in 2016

People within the model can die from specific diseases or from other causes. A disease file is created within the program to represent deaths from other causes. The following distributions are required by the population editor (Table 7).

Table 7 Summary of the parameters representing the distribution component

Distribution name	symbol	note
MalesByAgeByYear	$p_m(a)$	Input in year0 – probability of a male having age a
FemalesByAgeByYear	$p_f(a)$	Input in year0 – probability of a female having age a

Deaths from modelled diseases

The simulation models any number of specified diseases some of which may be fatal. In the start year the simulation's death model uses the diseases' own mortality statistics to adjust the probabilities of death by age and gender. In the start year the net effect is to maintain the same probability of death by age and gender as before; in subsequent years, however, the rates at which people die from modelled diseases will change as modelled risk factors change.

The risk factor model

The distribution of risk factors (RF) in the population is estimated using regression analysis stratified by both sex *S* = {male, female} and age group $A = \{0-4,\ 5-9,\ ...,\ 70-74,\ 75+\}$. The fitted trends are extrapolated to forecast the distribution of each RF category in the future. For each sex-and-age-group stratum, the set of cross-sectional, time-dependent, discrete distributions $D = \{p_k(t) \mid k = 1, \ldots N; t > 0\}$, is used to manufacture RF trends for individual members of the population. Each BMI is modelled as a continuous risk factor.

Continuous risk factors

In the case of a continuous RF, for each discrete distribution D there is a continuous counterpart. Let β denote the RF value in the continuous scale and let $f(\beta \mid A, S, t)$ be the probability density function of β for age group A A and sex S at time t. Then

$$p_k(t|A,S) = \int_{\beta \in k} f(\beta|A,S,t)\, d\beta. \qquad (0.4)$$

Equations (0.2) and (0.4) both refer to the same quantity. However, equation (0.4) uses the definition of the probability density function to express the age-and-sex-specific percentage of individuals in RF category *k* at time *t*. Equation (0.2) gives an estimate of this quantity using equation (0.1) for all $k = 0, \ldots, N$. The cumulative distribution function of β is

$$F(\beta|A,S,t) = \int_0^{\beta} f(\beta|A,S,t)\, d\beta. \qquad (0.5)$$

At time *t*, a person with sex S belonging to the age group A is said to be on the $p-$th percentile of this distribution if $F(\beta|A,S,t) = p/100$. Given the cross-sectional information from the set of distributions D, it is possible to simulate longitudinal trajectories by forming pseudo-cohorts within the population. A key requirement for these sets of longitudinal trajectories is that they reproduce the cross-sectional distribution of RF categories for any year with available data. The method adopted here and in our earlier work is based on the assumption that person's RF value changes throughout their lives in such a

way that they always have the same associated percentile rank. As they age, individuals move from one age group to another and their RF value changes so that they have the same percentile rank but of a different RF distribution. Crucially it meets the important condition that the cross-sectional RF distributions obtained by simulation match the RF distributions of the observed data.

The above procedure can be explained using the example of the NO_2 distribution. The NO_2 distributions are known for the population stratified by sex and age for all years of the simulation (by extrapolation of fitted model, see equation (0.1)). A person who is in age group A and who grows ten years older will at some time move into the next age group A' and will have a BMI that was described first by the distribution $f(\beta | A, S, t)$ and then at the later time t' by the distribution $f(\beta | A', S, t')$. If the BMI of that individual is on the $p-$th percentile of the BMI distribution, their BMI will change from β to β' so that

$$\beta = F^{-1}\left(\frac{p}{100} | A, S, t\right) \tag{0.6}$$

$$\beta' = F^{-1}\left(\frac{p}{100} | A', S, t'\right) \Rightarrow \beta' = F^{-1}\left(F(\beta | A, S, t) | A', S, t'\right) \tag{0.7}$$

Where F^{-1} is the inverse of the cumulative distribution function of β, which we model with a continuous uniform distribution within the RF categories (see **Table 6**). Equation (0.7) guarantees that the transformation taking the random variable β to β' ensures the correct cross-sectional distribution at time t'.

The microsimulation first generates individuals from the RF distributions of the set D and, once generated, grows the individual's RF in a way that is also determined by the set D. It is possible to implement equation (0.7) as a suitably fast algorithm.

Relative risks

Suppose that α is a risk factor state of some risk factor A and denote by $p_A(d | a, a, s)$ the incidence probability for the disease d given the risk state, α, the person's age, a, and gender, s. The relative risk ρ_A is defined by equation (0.7).

$$\begin{aligned} p_{\mathrm{A}}(d|\alpha, a, s) &= \rho_{\mathrm{A}|d}(\alpha|a, s)\, p_{\mathrm{A}}(d|\alpha_0, a, s) \\ \rho_{\mathrm{A}|d}(\alpha_0|a, s) &\equiv 1 \end{aligned} \tag{0.7}$$

Where α_0 is the zero risk state.

The incidence probabilities, as reported, can be expressed in terms of the equation,

$$\begin{aligned} p(d|a,s) &= \sum_{\alpha} p_{A}(d|\alpha,a,s)\pi_{A}(\alpha|a,s) \\ &= p_{A}(d|\alpha_0,a,s)\sum_{\alpha}\rho_{A|d}(\alpha|a,s)\pi_{A}(\alpha|a,s) \end{aligned} \quad (0.7)$$

Combining these equations allows the conditional incidence probabilities to be written in terms of known quantities

$$p(d|\alpha,a,s) = \rho_{A|d}(\alpha|a,s)\frac{p(d|a,s)}{\sum_{\beta}\rho_{A|d}(\beta|a,s)\pi_{A}(\alpha|a,s)} \quad (0.7)$$

Previous to any series of Monte Carlo trials the microsimulation program pre-processes the set of diseases and stores the *calibrated* incidence statistics $p_A(d|a_0,a,s)$. These incidence statistics are calibrated to national level data sets for both national level and local authority model simulations. In this project the risk factor distributions and incidence risks for England are used to calculate the calibrated risks.

Modelling diseases

Disease modelling relies heavily on the sets of incidence, mortality, survival, relative risk and prevalence statistics. In some cases where a data set is unavailable or not available is the specified form for the model, data has been approximated from the known sets of the data.

The microsimulation uses risk dependent incidence statistics and these are inferred from the relative risk statistics and the distribution of the risk factor within the population. In the simulation, individuals are assigned a risk factor trajectory giving their personal risk factor history for each year of their lives. Their probability of getting a particular risk factor related disease in a particular year will depend on their risk factor state in that year.

Once a person has a fatal disease (or diseases) their probability of survival will be controlled by a combination of the disease-survival statistics and the probabilities of dying from other causes. Disease survival statistics are modelled as age and gender dependent exponential distributions.

Mortality statistics

In any year, in some population, in a sample of N N people who have the disease a subset N_ω will die from the disease.

Mortality statistics record the cross sectional probabilities of death as a result of the disease – possibly stratifying by age

$$p_\omega = \frac{N_\omega}{N} \tag{0.8}$$

Within some such subset N_ω of people that die in that year from the disease, the distribution by year-of-disease is not usually recorded. This distribution would be most useful. Consider two important idealised, special cases

Suppose the true probabilities of dying in the years after some age a_0 are $\{p_{\omega 0}, p_{\omega 1}, p_{\omega 2}, p_{\omega 3}, p_{\omega 4}\}$. The probability of being alive after N years is simply that you don't die in each year

$$p_{survive}\left(a_0 + N\right) = \left(1 - p_{\omega 0}\right)\left(1 - p_{\omega 1}\right)\left(1 - p_{\omega 2}\right)..\left(1 - p_{\omega N-1}\right) \tag{0.9}$$

Survival rates

It is common practice to describe survival in terms of a survival rate R . supposing an exponential death-distribution. In this formulation the probability of surviving t years from some time t_0 is given as

$$p_{\text{survival}}\left(t\right) = 1 - R\int_0^t due^{-Ru} = e^{-Rt} \tag{0.10}$$

For a time period of 1 year

$$\begin{gathered} p_{survival}\left(1\right) = e^{-R} \\ \Rightarrow \\ R = -\ln\left(p_{survival}\left(1\right)\right) = -\ln\left(1 - p_\omega\right) \end{gathered} \tag{0.11}$$

For a time period of, for example, 4 years,

$$p_{\text{survival}}\left(t = 4\right) = 1 - R^{-1}\int_0^4 due^{-Ru} = e^{-4R} = \left(1 - p_\omega\right)^4 \tag{0.12}$$

In short, the Rate is minus the natural log of the 1-year survival probability.

The survival models

For any potentially terminal disease the model can use any of the three survival models, numbered ((0, 1, 2)). The parameters describing these models are given below.

Survival model 0

A single probability of dying $\{p_{\omega 0}\}$, where $p_{\omega 0}$ is valid for all years. Given the 1-year survival probability $p_{survival}(1)$

The model uses 1 parameter ((R))

$$R = -\ln\left(p_{survival}(1)\right) \tag{0.13}$$

Survival model 1

Two different probabilities of dying $\{p_{\omega 0}, p_{\omega 1}\}$, where $p_{\omega 0}$ is valid for the first year; $p_{\omega 1}$ thereafter. The model uses two parameters $(p_1,\ R)$. Given the 1-year survival probability $p_{survival}(1)$ and the 5-year survival probability $p_{survival}(5)$

$$\begin{aligned} p_1 &= 1 - p_{survival}(1) \\ R &= -\frac{1}{4}\ln\left(\frac{p_{survival}(5)}{p_{survival}(1)}\right) \end{aligned} \tag{0.14}$$

Survival model 2

Three different probabilities of dying $\{p_{\omega 0}, p_{\omega 1}, p_{\omega 5}\}$, where $p_{\omega 0}$ is valid for the first year; $p_{\omega 1}$ for the second to the fifth year; $p_{\omega 5}$ thereafter. The model uses three parameters $(p_1,\ R,\ R_{>5})$

Given the 1-year survival probability $p_{survival}(1)$ and the 5-year survival probability $p_{survival}(5)$

$$\begin{aligned} p_1 &= 1 - p_{survival}(1) \\ R &= -\frac{1}{4}\ln\left(\frac{p_{survival}(5)}{p_{survival}(1)}\right) \\ R_{>5} &= -\frac{1}{5}\ln\left(\frac{p_{survival}(10)}{p_{survival}(5)}\right) \end{aligned} \tag{0.15}$$

Remember that different probabilities will apply to different age and gender groups. Typically the data might be divided into 10 year age groups.

Approximating missing disease statistics

A number of tools have been developed in the model in order to compute missing disease statistics data such as incidence or prevalence.

Approximating survival data from mortality and prevalence

An example is provided here with a standard life-table analysis for a disease d.

Consider the 4 following states:

state	Description
0	alive without the disease
1	alive with the disease
2	dead from the disease
3	dead from other diseases

p_{ik} is the probability of the disease incidence at aged k

$p_{\omega k}$ is the probability of dying from the at aged k

$p_{\varpi k}$ is the probability of dying other than from the disease at aged k

The state transition matrix is constructed as follows

$$\begin{bmatrix} p_0(k+1) \\ p_1(k+1) \\ p_2(k+1) \\ p_3(k+1) \end{bmatrix} = \begin{bmatrix} (1-p_{\varpi k})(1-p_{ik}) & (1-p_{\varpi k}-p_{\omega k})\,p_{\alpha k} & 0 & 0 \\ (1-p_{\varpi k})\,p_{ik} & (1-p_{\varpi k}-p_{\omega k})(1-p_{\alpha k}) & 0 & 0 \\ 0 & p_{\omega k} & 1 & 0 \\ p_{\varpi k} & p_{\varpi k} & 0 & 1 \end{bmatrix} \begin{bmatrix} p_0(k) \\ p_1(k) \\ p_2(k) \\ p_3(k) \end{bmatrix} \tag{0.16}$$

The disease mortality equation is that for state-2,

$$p_2(k+1) = p_{\omega k}\,p_1(k) + p_2(k) \tag{0.17}$$

The probability of dying from the disease in the age interval [*k*, *k*+1] is $p_{\omega k}\,p_1(k)$ - this is otherwise the (cross-sectional) disease mortality, $p_{mor}(k)$. $p_1(k)$ is otherwise known as the disease prevalence, $p_{pre}(k)$. Hence the relation

$$p_{\omega k} = \frac{p_{mor}(k)}{p_{pre}(k)} \qquad (0.18)$$

For exponential survival probabilities the probability of dying from the disease in the age-interval [*k*, *k*+1] is denoted by $p_{\omega k}$ and is given by the formula

$$p_{\omega k} = 1 - e^{-R_k} \quad \Rightarrow \quad R_k = -\ln\left(1 - p_{\omega k}\right) \qquad (0.19)$$

When, as is the case for most cancers, these survival probabilities are known the microsimulation will use them, when they are not known or are too old to be any longer of any use, the microsimulation uses survival statistics inferred from the prevalence and mortality statistics (equation (0.18)). An alternative derivation equation (0.18) is as follows. Let N_k be the number of people in the population aged k and let n_k be the number of people in the population aged k with the disease. Then, the number of deaths from the disease of people aged k can be given in two ways: as $p_{\omega k} n_k$ and, equivalently, as $p_{mor}(k) N_k$. Observing that the disease prevalence is n_k / N_k leads to the equation

$$\begin{aligned} p_{\omega k} n_k &= p_{mor}(k) N_k \\ p_{pre}(k) &= \frac{n_k}{N_k} \\ \Rightarrow p_{\omega k} &= \frac{p_{mor}(k)}{p_{pre}(k)} \end{aligned} \qquad (0.20)$$

Approximating disease incidence from prevalence

The algorithm estimates the probability of contracting a disease given age and sex, $\hat{p}(d \mid a, s)$ from prevalence rates, survival rates and mortality rates.

Step 1: State transition matrix of the algorithm

$$\begin{pmatrix} p_{\bar{d}}(a+1 \mid s) \\ p_{d1}(a+1 \mid s) \\ p_d(a+1 \mid s) \\ p_{dead}(a+1 \mid s) \end{pmatrix} = \begin{pmatrix} (1 - p_{\bar{w}}(a \mid s))(1 - \hat{p}(d \mid a, s)) & 0 & 0 & 0 \\ (1 - p_{\bar{w}}(a \mid s))\hat{p}(d \mid a, s) & 0 & 0 & 0 \\ 0 & 1 - p_{w1+\bar{w}1}(a \mid s) & 1 - p_{w+\bar{w}}(a \mid s) & 0 \\ p_{\bar{w}}(a \mid s) & p_{w1+\bar{w}1}(a \mid s) & p_{w+\bar{w}}(a \mid s) & 1 \end{pmatrix} \begin{pmatrix} p_{\bar{d}}(a \mid s) \\ p_{d1}(a \mid s) \\ p_d(a \mid s) \\ p_{dead}(a \mid s) \end{pmatrix}$$

(0.21)

The probability of being in a set of states:

S_0	$p_{\bar{d}}(a \mid s)$	The probability of being alive without disease at age a

S_1	$p_{d1}(a \mid s)$	The probability of being alive with new disease (contracting within a year) at age a
S_2	$p_d(a \mid s)$	The probability of being alive with old disease at age a
S_3	$p_{dead}(a \mid s)$	The probability of being dead for any reason (from the disease or other reasons) at age a

$\hat{p}(d \mid a, s)$ The estimated incidence probability at age of a given sex type s .

$p_{\bar{w}}(a \mid s)$ The probability of dying from other causes at age of a given sex type s.

$p_{w1+\bar{w}1}(a \mid s)$ The probability of dying from any reason within the first years of contracting the disease at the age of a given sex type s.

$p_{w+\bar{w}}(a \mid s)$ The probability of dying from any reasons after the first years of contracting the disease at the age a given sex type s.

$p_{survival1st}(a \mid s)$ The probability of surviving the first year after contracting the disease at the age of a given sex type s.

$p_{survival1}(a \mid s)$ The probability of surviving the year at the age of a given sex type s.

Step 2: The prevalence for a particular age group
Estimated prevalence rate can be expressed by,

$$\hat{P}_{pre\_mean}(agegroup \mid s) = \frac{\sum_{\min\_a}^{\max\_a} \hat{P}_{pre}(a \mid s) \cdot \pi(a \mid s)}{\sum_{\min\_a}^{\max\_a} \pi(a \mid s)} \quad (0.22)$$

where

$$\hat{P}_{pre}(a \mid s) = \frac{p_d(a \mid s) + p_{d1}(a \mid s)}{p_d(a \mid s) + p_{d1}(a \mid s) + p_{\bar{d}}(a \mid s)} \quad (0.23)$$

where $\min\_a$ is the youngest age in that age group and $\max\_a$ the oldest. $\pi(a \mid s)$ is the population distribution stratified by age given sex.

Step 3: Regression
We have two algorithms to find the optimum value of $\hat{p}(d \mid a, s)$: simplex algorithm and cauchy algorithm. Simplex algorithm finds an optimum set of incidence rates of all age groups by minimising the

distance between the estimated global prevalence rate and the actual global prevalence rate, shown in (1.37). We use simplex algorithm for most diseases as it is faster.

$$\underset{set(\hat{p}(d|a,s))}{\arg\min}\ S = \underset{set(\hat{p}(d|a,s))}{\arg\min}\ S\left(\sum_{age\_group}(P_{pre\_mean}(agegroup\,|\,s) - \hat{P}_{pre\_mean}(agegroup\,|\,s))\right) \quad (0.24)$$

Cauchy algorithm finds an optimum incidence rate for each individual age group by minimising the distance between the estimated prevalence rate and the actual prevalence rate of the age group, shown in (0.25). We use Cauchy algorithm for diseases which are associated to certain age groups, e.g., dementia which is only associated to people older than 60.

$$\underset{\hat{p}(d|a,s)}{\arg\min}\, S = \underset{\hat{p}(d|a,s)}{\arg\min}\, S\left(P_{pre\_mean}(agegroup\,|\,s) - \hat{P}_{pre\_mean}(agegroup\,|\,s)\right) \quad (0.26)$$

References

1. Statistics OfN. Population Estimates for UK, England and Wales, Scotland and Northern Ireland: mid-2016. 2017.
2. Office for National Statistics. Deaths registered in England and Wales: 2015. 2016.
3. Scotland NRo. Deaths, by sex and single year of age, Scotland 1974 to 2016. Deaths Time Series Data2017.
4. Agency NISaR. Deaths by single year of age, 1955 to 2015. 2017.
5. Ahern AL, Aveyard PN, Halford JC, Mander A, Cresswell L, Cohn SR, et al. Weight loss referrals for adults in primary care (WRAP): protocol for a multi-centre randomised controlled trial comparing the clinical and cost-effectiveness of primary care referral to a commercial weight loss provider for 12 weeks, referral for 52 weeks, and a brief self-help intervention [ISRCTN82857232]. BMC public health. 2014;14(1):620.
6. British Heart Foundation. Cardiovascular Disease Statistics 2014. 2015.
7. Arthritis Research UK. Musculoskeletal Calculator 2016. Available from: http://www.arthritisresearchuk.org/arthritis-information/data-and-statistics/musculoskeletal-calculator.aspx.
8. Zheng H, Chen C. Body mass index and risk of knee osteoarthritis: systematic review and meta-analysis of prospective studies. BMJ open. 2015;5(12):e007568.
9. British Heart Foundation. Stroke Statistics 2009. 2009.
10. World Obesity Federation. Relative risk Assessments IASO; Prepared for DYNAMO-HIA project. Available from: http://www.worldobesity.org/site_media/uploads/Appendix_Relative_Risk_Assessments_IASO.pdf; http://www.worldobesity.org/what-we-do/policy-prevention/projects/eu-projects/dynamohiaproject/estimatesrrperunitbmi/.
11. Statistics OfN. Cancer registration statistics, England. ONS; 2015.
12. Smolina K, Wright FL, Rayner M, Goldacre MJ. Determinants of the decline in mortality from acute myocardial infarction in England between 2002 and 2010: linked national database study. Corrected data on incidence and mortality in 2013 at http://www.bmj.com/content/347/bmj.f7379.abstract. BMJ. 2012;344:d8059.
13. Dr. Craig Currie at Cardiff University.
14. NHS Digital. National Diabetes Audit 2015/2016 NHS Digital2017. Available from: http://www.content.digital.nhs.uk/catalogue/PUB23241.
15. Health and Social Care Information Centre. Health Survey for England - 2014 - Trend Tables. Available from http://www.hscic.gov.uk/catalogue/PUB19297. 2015.
16. Office for National Statistics. Cancer Survival in England: adults diagnosed between 2011 and 2015 and followed up to 2016. 2017.
17. National Health Service. Health Investment Networks 2012-13 Programme Budgeting data 2016. Available from: https://www.networks.nhs.uk/nhs-networks/health-investment-network/news/2012-13-programme-budgeting-data-is-now-available.
18. International Diabetes F. IDF Diabetes Atlas 6th Edition 2014 2014. Available from: https://www.idf.org/sites/default/files/Atlas-poster-2014_EN.pdf.
19. Oxford Economics. The economic cost of arthritis for the UK economy - Final report. 2010.
20. Roberta Ara AW. NICE DSU technical support document 12: the use of health state utility values in decision models. 2011.
21. Patrick W. Sullivan JFS, Mark J. Sculpher, Vahram Ghushchyan. Catalogue of EQ-5D Scores for the United Kingdom. Medical Decision Making. 2011;31(6):800-4.
22. Laires PA, Ejzykowicz F, Hsu TY, Ambegaonkar B, Davies G. Cost-effectiveness of adding ezetimibe to atorvastatin vs switching to rosuvastatin therapy in Portugal. J Med Econ. 2015;18(8):565-72.
23. Conner-Spady BL, Marshall DA, Bohm E, Dunbar MJ, Loucks L, Al Khudairy A, et al. Reliability and validity of the EQ-5D-5L compared to the EQ-5D-3L in patients with osteoarthritis referred for hip and knee replacement. Qual Life Res. 2015;24(7):1775-84.
24. Romanus D, Kindler HL, Archer L, Basch E, Niedzwiecki D, Weeks J, et al. Does health-related quality of life improve for advanced pancreatic cancer patients who respond to

gemcitabine? Analysis of a randomized phase III trial of the cancer and leukemia group B (CALGB 80303). J Pain Symptom Manage. 2012;43(2):205-17.
25. Rivero-Arias O, Ouellet M, Gray A, Wolstenholme J, Rothwell PM, Luengo-Fernandez R. Mapping the modified Rankin scale (mRS) measurement into the generic EuroQol (EQ-5D) health outcome. Med Decis Making. 2010;30(3):341-54.
