# Supplementary material for: Cost-effectiveness of bariatric surgery and non-surgical weight management programmes for adults with severe obesity: a decision analysis model
Source: Int J Obes (Lond). 2021 Jun 4;45(10):2179–90. doi: 10.1038/s41366-021-00849-8 (PMC8455321; doi:10.1038/s41366-021-00849-8)
Supplement: Supplementary file 2 — Intervention costs [file 41366_2021_849_MOESM2_ESM.docx]

## Supplementary materials: Full break down of intervention costing.

## Table 1 Bariatric surgery costs

|  | **Year 1** | **Year 2** | **Year 3** | **Year 4** | **Year 5** | **Year 6** | **Year 7** | **Year 8** | **Year 9** | **Year 10** | **Annual costs from year 11 to 30** | **Total costs year 1 to 10** | **Total costs over a lifetime (undiscounted)** |
| --- | --- | --- | --- | --- | --- | --- | --- | --- | --- | --- | --- | --- | --- |
| **Surgery cost** | £4,886 | £0 | £0 | £0 | £0 |  |  |  |  |  |  | £4,886 |  |
| **Direct staff time** | £2,387 | £718 | £375 | £150 | £154 | £109 | £109 | £109 | £109 | £109 | £109 | £4,329 |  |
| **Rentals** | £6 | £39 | £36 | £0 | £0 |  |  |  |  |  |  | £81 |  |
| **Materials** | £5 | £0 | £0 | £0 | £0 |  |  |  |  |  |  | £5 |  |
| **Blood tests** | £356 | £178 | £178 | £178 | £178 | £178 | £178 | £178 | £178 | £178 | £178 | £1,958 |  |
| **Vitamins and minerals** | £248 | £248 | £248 | £248 | £248 | £248 | £248 | £248 | £248 | £248 | £248 | £2,485 |  |
| **Complications** | £282 | £293 | £0 | £0 | £0 |  |  |  |  |  |  | £575 |  |
| **Revision surgery** | £83 | £83 | £83 | £83 | £83 | £83 | £83 | £83 | £83 | £83 |  | £831 |  |
| **Total** | **£8,253** | **£1,559** | **£921** | **£659** | **£663** | **£619** | **£619** | **£619** | **£619** | **£619** | **£536** | **£15,150** | **£25,862** |

## Table 2 WMP2 costs

|  | **Year 1** | **Year 2** | **Year 3** | **Year 4** | **Year 5** | **Total costs** |
| --- | --- | --- | --- | --- | --- | --- |
| **Direct staff time** | £416 | £116 | £150 | £204 | £111 | £997 |
| **Rentals** | £199 | £36 | £36 | £0 | £0 | £271 |
| **Materials** | £5 | £0 | £0 | £0 | £0 | £5 |
| **Meal replacement costs** | £134 | £0 | £0 | £0 | £0 | £134 |
| **Total** | **£754** | **£152** | **£186** | **£204** | **£111** | **£1,407** |

## Table 3 VLCD costs

|  | **Year 1** | **Year 2** | **Year 3** | **Year 4** | **Total** |
| --- | --- | --- | --- | --- | --- |
| **Direct staff time** | £458 | £193 | £53 | £9 | £713 |
| **Rentals** | £267 | £75 | £8 | £0 | £349 |
| **Materials** | £5 | £0 | £0 | £0 | £5 |
| **Meal replacement costs** | £1,163 | £0 | £0 | £0 | £1,163 |
| **Total** | **£1,893** | **£268** | **£60** | **£9** | **£2,230** |

## Table 4 WMP1 costs

|  | **Year 1** | **Year 2** | **Year 3** | **Year 4** | **Total** |
| --- | --- | --- | --- | --- | --- |
| **Direct staff time** | £406 | £193 | £53 | £9 | £661 |
| **Rentals** | £208 | £75 | £8 | £0 | £290 |
| **Materials** | £5 | £0 | £0 | £0 | £5 |
| **Meal replacement costs** | £0 | £0 | £0 | £0 | £0 |
| **Total** | **£619** | **£268** | **£60** | **£9** | **£956** |

## Table 5 Costs and utilities of surgery complications

| **Year 1** | | | | | | | | | |
| --- | --- | --- | --- | --- | --- | --- | --- | --- | --- |
| **Procedure** | **Utility value** | **Source** | **Utility weight** | **Rates of complications** | **Source** | **Rate of complications, rescaled** | **Utility weight**  *** rate of complication** | **Total costs of complications** | **Total cost * rate of complication** |
| Internal hernia | 0.6800 | Canavan 2015 [1] | 0.7907 | 1% | Puzziferri et al. 2014 [2] | 6% | 0.0488 | £2,951.81 | £59.04 |
| Incisional hernia | 0.6800 | Canavan 2015 [1] | 0.7907 | 1% | Puzziferri et al. 2014 [2] | 6% | 0.0488 | £2,951.81 | £59.04 |
| Marginal ulcer | 0.6800 | Canavan 2015 [1] | 0.7907 | 1% | Puzziferri et al. 2014 [2] | 6% | 0.0488 | £882.99 | £8.83 |
| Anaemia | 0.7640 | NICE 2015 [3] | 0.8884 | 2% | Puzziferri et al. 2014 [2] | 12% | 0.1097 | £172.40 | £3.45 |
| Iron deficiency requiring transfusion | 0.7640 | NICE 2015 [3] | 0.8884 | 2% | Puzziferri et al. 2014 [2] | 12% | 0.1097 | £172.40 | £3.45 |
| Operational revision rates for abdominal pain | 0.6800 | Canavan 2015 [1] | 0.7907 | 0.10% | Puzziferri et al. 2014 [2] | 0.62% | 0.0049 | £1,691.87 | £1.69 |
| Operational revision rates for non-healing ulcer | 0.6800 | Canavan 2015 [1] | 0.7907 | 0.10% | Puzziferri et al. 2014 [2] | 0.62% | 0.0049 | £2,076.60 | £2.08 |
| Gastrointestinal bleeding rate | 0.7350 | Campbell 2015 [4] | 0.8547 | 1% | Puzziferri et al. 2014 [2] | 6% | 0.0528 | £1,193.61 | £11.94 |
| Cholecystectomy year 1 | 0.6400 | Brazzelli 2014 [5] | 0.7442 | 8% | Picot 2009 [6] | 49% | 0.3675 | £2,442.12 | £195.37 |
| **Average utility weighted value across all health states** |  |  |  | 16% |  | 100% | **0.7958** |  |  |
| **Year 2** | | | | | | | | | |
| **Procedure** | **Utility value** | **Source** | **Utility weight** | **Rates of complications** | **Source** | **Rate of complications, rescaled** | **Utility weight * rate of complication** | **Total costs of complications** | **Total cost * rate of complication** |
| Cholecystectomy year 2 | 0.6400 | Brazzelli 2014 [5] | 0.7442 | 12% | Picot 2009 [6] | 100% | 0.7442 | £2,442.12 | £293.05 |
| **Average utility weighted value across all health states** |  |  |  | 12% |  | 100% | **0.7442** |  |  |

**REFERENCES:**

| [1] | Canavan C, West J, Card T. Change in quality of life for patients with irritable bowel syndrome following referral to a gastroenterologist: A cohort study*. PLoS ONE* 2015;**10** e0139389. |
| --- | --- |
| [2] | Puzziferri N, Roshek ITB, Mayo HG, Gallagher R, Belle SH, Livingston EH. Long-term follow-up after bariatric surgery: A systematic review*. JAMA* 2014;**312:**934-42. |
| [3] | *Costing statement: Blood transfusion. Implementing the NICE guideline on blood transfusion (NG24)*: National Institute for Health and Care Excellence; 2015. <https://www.nice.org.uk/guidance/ng24/resources/costing-statement-pdf-2177158141> [Accessed May 2018] |
| [4] | Campbell HE, Stokes EA, Bargo D, Logan RF, Mora A, Hodge R, et al. Costs and quality of life associated with acute upper gastrointestinal bleeding in the UK: cohort analysis of patients in a cluster randomised trial*. BMJ Open* 2015;**5:**e007230. |
| [5] | Brazzelli M, Cruickshank M, Kilonzo M, Ahmed I, Stewart F, McNamee P, et al. Clinical effectiveness and cost-effectiveness of cholecystectomy compared with observation/conservative management for preventing recurrent symptoms and complications in adults presenting with uncomplicated symptomatic gallstones or cholecystitis: A systematic review and economic evaluation*. Health Technol Assess* 2014;**18:**1-101. |
| [6] | Picot J, Jones J, Colquitt JL, Gospodarevskaya E, Loveman E, Baxter L, et al. The clinical effectiveness and cost-effectiveness of bariatric (weight loss) surgery for obesity: A systematic review and economic evaluation*. Health Technol Assess* 2009;**13:**41. |
