# Supplementary material for: Cost-effectiveness of bariatric surgery and non-surgical weight management programmes for adults with severe obesity: a decision analysis model
Source: Int J Obes (Lond). 2021 Jun 4;45(10):2179–90. doi: 10.1038/s41366-021-00849-8 (PMC8455321; doi:10.1038/s41366-021-00849-8)
Supplement: Supplementary file 3 — Sensitivity analyses [file 41366_2021_849_MOESM3_ESM.pdf]

Table S1 Cost-effectiveness sensitivity analyses around time horizon and discount rate

Intervention	Intervention cost (£m/100k population)	Obesity disease cost (£m/100k population)	Total cost (£m/100k population)	Total QALY per 100k population	Multiple treatment comparison: Inc. Costs	Inc. QALYS	ICER (vs. next best alternative)	ICER vs. baseline
**0% Discounting of costs and QALYs**								
Baseline	£0	£3,714	£3,714	1,345,761	Dominated	Dominated	Dominated	--
WMP1	£94	£3,603	£3,697	1,370,416	--	--	--	Dominant
VLCD added to WMP1	£220	£3,600	£3,820	1,371,637	Dominated	Dominated	Dominated	£4,110
WMP2	£137	£3,581	£3,718	1,374,914	£21	4,498	£4,708	£146
Look AHEAD	£928	£3,522	£4,450	1,386,615	Ext Dom.	Ext Dom.	Ext Dom.	£18,014
RYGB Surgery	£2,257	£2,899	£5,156	1,532,976	£1,437	158,062	£9,094	£7,701
**3.5% Discounting of costs and QALYs**								
Baseline	£0	£2,151	£2,151	930,221				
WMP1	£93	£2,091	£2,184	943,960	£33	13,739	£2,379	£2,379
VLCD added to WMP1	£219	£2,089	£2,308	944,781	Dominated	Dominated	Dominated	£10,774
WMP2	£132	£2,079	£2,211	946,423	Ext Dom.	Ext Dom.	Ext Dom.	£3,726
Look AHEAD	£842	£2,050	£2,892	952,437	Ext Dom.	Ext Dom.	Ext Dom.	£33,354
RYGB Surgery	£1,798	£1,736	£3,534	1,027,562	£1,350	83,602	£16,151	£14,207
**6% Discounting of costs and QALYs**								
Baseline	£0	£1,556	£1,556	751,490	--	--	--	
WMP1	£92	£1,515	£1,607	761,029	£51	9,539	£5,362	£5,362
VLCD added to WMP1	£218	£1,513	£1,731	761,679	Dominated	Dominated	Dominated	£17,245
WMP2	£129	£1,507	£1,636	762,706	Ext Dom.	Ext Dom.	Ext Dom.	£7,166
Look AHEAD	£790	£1,489	£2,279	766,619	Ext Dom.	Ext Dom.	Ext Dom.	£47,801
RYGB Surgery	£1,605	£1,285	£2,889	815,451	£1,282	54,422	£23,565	£20,851
**5 year time horizon**								
Baseline	£0	£388	£388	319,023	--	--	--	--
WMP1	£94	£384	£477	320,030	£89	1,007	£88,689	£88,689
WMP2	£135	£383	£518	320,037	Ext Dom.	Ext Dom.	Ext Dom.	£128,511
VLCD added to WMP1	£220	£383	£603	320,256	£125	226	£555,265	£174,209
Look AHEAD	£641	£383	£1,024	320,295	Ext Dom.	Ext Dom.	Ext Dom.	£500,024
RYGB Surgery	£1,172	£366	£1,537	320,665	£935	409	£2,285,770	£700,171

Intervention	Intervention cost (£m/100k population)	Obesity disease cost (£m/100k population)	Total cost (£m/100k population)	Total QALY per 100k population	Multiple treatment comparison: Inc. Costs	Inc. QALYS	ICER (vs. next best alternative)	ICER vs. baseline
**10 year time horizon**								
Baseline;	£0	£804	£804	575,958	--	--	--	--
WMP1	£94	£791	£884	579,578	£80	3,620	£21,975	£21,975
VLCD added to WMP1	£220	£789	£1,009	580,047	Dominated	Dominated	Dominated	£50,000
WMP2	£135	£788	£923	580,099	Ext Dom.	Ext Dom.	Ext Dom.	£28,551
Look AHEAD	£889	£787	£1,676	580,650	Ext Dom.	Ext Dom.	Ext Dom.	£185,688
RYGB Surgery	£1,421	£732	£2,153	592,597	£1,269	13,019	£97,449	£81,029
**20 year time horizon**								
Baseline	£0	£1,732	£1,732	931,620	--	--	--	--
VLCD added to WMP1	£220	£1,686	£1,905	943,556	Dominated	Dominated	Dominated	£14,542
WMP1	£94	£1,688	£1,781	943,732	£49	12,112	£4,077	£4,077
WMP2	£135	£1,679	£1,814	944,768	Ext Dom.	Ext Dom.	Ext Dom.	£6,233
Look AHEAD	£889	£1,660	£2,549	949,072	Ext Dom.	Ext Dom.	Ext Dom.	£46,854
RYGB Surgery	£1,767	£1,470	£3,237	1,000,384	£1,456	56,652	£25,697	£21,889
